# Visual interpretable MRI fine grading of meniscus injury for intelligent assisted diagnosis and treatment

**DOI:** 10.1038/s41746-024-01082-z

**Published:** 2024-04-15

**Authors:** Anlin Luo, Shuiping Gou, Nuo Tong, Bo Liu, Licheng Jiao, Hu Xu, Yingchun Wang, Tan Ding

**Affiliations:** 1 Key Laboratory of Intelligent Perception an Image Understanding of Ministry of Education, School of Artificial Intelligence, Xidian University, 710071 Xi’an, China; 2 AI-based Big Medical lmaging Data Frontier Research Center, Academy of Advanced Interdisciplinary Research, Xidian University, 710071 Xi’an, Shaanxi China; 3 Xijing Orthopaedics Hospital, The Fourth Military Medical University, 710032 Xi’an, Shaanxi China

**Keywords:** Diseases, Experimental models of disease, Diagnosis

## Abstract

Meniscal injury represents a common type of knee injury, accounting for over 50% of all knee injuries. The clinical diagnosis and treatment of meniscal injury heavily rely on magnetic resonance imaging (MRI). However, accurately diagnosing the meniscus from a comprehensive knee MRI is challenging due to its limited and weak signal, significantly impeding the precise grading of meniscal injuries. In this study, a visual interpretable fine grading (VIFG) diagnosis model has been developed to facilitate intelligent and quantified grading of meniscal injuries. Leveraging a multilevel transfer learning framework, it extracts comprehensive features and incorporates an attributional attention module to precisely locate the injured positions. Moreover, the attention-enhancing feedback module effectively concentrates on and distinguishes regions with similar grades of injury. The proposed method underwent validation on FastMRI_Knee and Xijing_Knee dataset, achieving mean grading accuracies of 0.8631 and 0.8502, surpassing the state-of-the-art grading methods notably in error-prone Grade 1 and Grade 2 cases. Additionally, the visually interpretable heatmaps generated by VIFG provide accurate depictions of actual or potential meniscus injury areas beyond human visual capability. Building upon this, a novel fine grading criterion was introduced for subtypes of meniscal injury, further classifying Grade 2 into 2a, 2b, and 2c, aligning with the anatomical knowledge of meniscal blood supply. It can provide enhanced injury-specific details, facilitating the development of more precise surgical strategies. The efficacy of this subtype classification was evidenced in 20 arthroscopic cases, underscoring the potential enhancement brought by intelligent-assisted diagnosis and treatment for meniscal injuries.

## Introduction

The knee joints represent intricate articulations within the human body, playing a pivotal role in multi-directional movements such as weight-bearing, lower limb flexion, and extension. Notably, knee joints are highly susceptible to injury, with meniscal injuries constituting the most prevalent type, accounting for approximately 50% of all cases^1^. Meniscal injuries can lead to pain, swelling, restricted knee mobility, significantly curtailing a patient’s physical activity. Timely and accurate diagnosis of meniscus is crucial for preserving meniscus function. Presently, magnetic resonance imaging (MRI) stands as the primary diagnostic modality due to its high tissue resolution.

To ensure appropriate treatment of meniscal injuries, precise grading methods based on knee MRI have been proposed to distinctly assess the injury severity^2–4^. Commonly utilized clinical grading systems for meniscal injuries include the Fischer^5^ and Mink^6^ criteria. Fischer’s grading system is presented in Table 1. However, an increasing number of doctors are questioning the practice of making the diagnosis and the choice of surgical approach with subjective grading based on MRI, because doctors have different cognition of qualitative grading criteria and lack of repeatability. Moreover, meniscus occupies a small proportion in the entire knee joint, and the diversity and complexity of injury types pose challenges in accurately quantifying the severity of meniscal injuries. This limitation impedes the effective application of clinical diagnosis and treatment.

**Table 1:** MRI description of Fischer grading criteria

Grade 0	Normal, there is no high signal inside;
Grade 1	Manifested as flak-like or circular fat compression sequence with the high signal image in the meniscus, which did not reach the articular surface margin of the meniscus;
Grade 2	Presented as horizontal or oblique stripe lipid pressure sequence with high signal image, and the joint capsule can be reached if the meniscus articular surface is not reached;
Grade 3	Manifested as a high signal image of the lipid pressure sequence within the meniscus reaching the articular surface margin.

Moreover, in Fischer’s Grade 2 and Grade 3 meniscal injuries, partial or total resection stands as a common treatment approach. However, the current practice lacks a universally accepted clinical standard for determining the extent of resection. Inadequate resection may result in persistent pain and associated symptoms, while excessive resection brings accelerated joint degeneration. To preserve maximal meniscal function, a minimal suture-based resection or suture treatment plan should be performed on the injured meniscus. The determination of resection or suturing extents depends not only on the grading outcomes, but also on considerations of the vascular supply and the intrinsic healing potential of the injured region, pivotal for restoring meniscal functionality. Regrettably, existing grading methodologies fall short of accurately quantifying injury severity, analyzing signal distribution within the injured area, and elucidating the impact of vascular supply on meniscus injuries. In response to the above challenges, the grading methods for diagnosis, and treatment of knee meniscal injury assisted by artificial intelligence technology are developed.

Henceforth, numerous methods have been applied to the research of meniscus, encompassing segmentation^7–9^ and reconstruction^10^ of the meniscus region, along with endeavors in diagnosing and grading meniscal injuries, as well as investigating the mechanisms underlying meniscal injuries^11^. While these published methodologies have demonstrated promising outcomes, refining treatment accuracy necessitates a heightened focus on the fine grading and visual interpretation of meniscal injuries. Through a retrospective study, previously proposed meniscus grading methods have been categorized into traditional grading methods and deep learning approaches.

Traditional diagnostic methods for meniscus injuries can be categorized into supervised and unsupervised ones^12^. Supervised techniques, exemplified by Boniatis I et al. employed the region-growing method to segment the meniscus region from MRI. Subsequently, computerized image processing techniques were utilized to extract an array of texture features and spatial variations in pixel intensity. A classification system based on a Bayesian classifier was designed to distinguish normal meniscus from degraded meniscus^13^. Another supervised approach by Cemal K et al. involved edge detection filtering using histogram and statistical segmentation to precisely locate the knee meniscus. Then the meniscus region was analyzed by modifying the intensity distribution of the statistical model to detect meniscus tears^14^. Unsupervised ones such as Saygili A and Albayrak S et al. proposed methods for detecting and grading meniscus injuries using knee MRI. They employed Fuzzy-*C* means and orientation gradient histogram method, respectively^15,16^. While these methods demonstrated certain capabilities in identifying meniscal injuries, they were often confined to binary injury classification and lacked the precision required in diagnostic outcomes. Furthermore, most of these approaches were semi-automatic, requiring human intervention, thereby introducing potential subjectivity.

The subjectivity of manually extracted features will affect the diagnosis result of meniscus injuries, while just deep learning methods belong to end-to-end, which can effectively circumvent human intervention. Consequently, some methods of meniscus injury diagnosis based on deep learning have been proposed^17^. For instance, Couteaux V et al. trained a mask region-based convolutional neural network (R-CNN) to explicitly localize normal and torn menisci. This network was bolstered through ensemble aggregation and integrated into a shallow convnet for tear orientation classification. This study realized the problem of automatically detecting meniscal injury and classifying of tear direction^18^. Similarly, Roblot V et al. also constructed a classification network for meniscal tears for similar problems and the same public dataset^19^. In addition, Bien et al. leveraged the deep convolutional neural network model MRNET, utilizing 1130 instances for training and 120 for validation. This model aimed at grading meniscal injuries, achieving an area under the receiver operating characteristic curve of 0.847^20^. Pedoia et al. also performed more detailed injury grading on this basis, augmented the training set by a factor of 10 using the image amplification method, and used 3D-CNN to recognize meniscal injury after 2D-UNet segmentation. Meniscus injuries were classified into normal, mild-moderate and severe WORMS in 3D-CNN, with accuracy of 0.81, 0.78 and 0.75, respectively^21^. Deep learning methods for the diagnosis of knee meniscus injury can be automated analysis, but the research objectives predominantly remain within binary (with or without) or ternary (normal, mild or severe) grading. Despite enabling automated analysis, the above-mentioned methods may fall short in meeting the requisite accuracy and explanatory depth demanded by clinical practice.

**Fig. 1:**
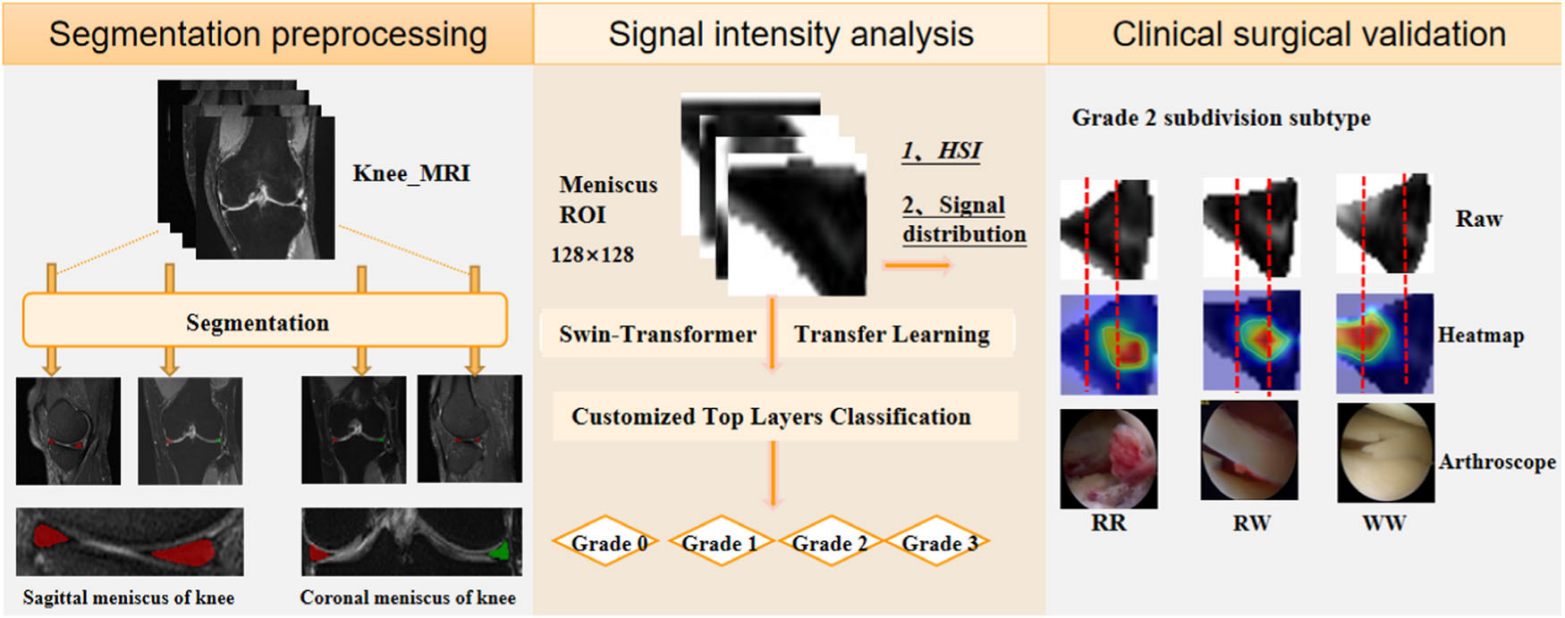
Schematic representation of the workflow for meniscal injury intelligent grading system. The segmentation preprocessing phase automates the segmentation of the meniscal region. Signal intensity analysis facilitates quantitative assessment of injury signals and enables refined automatic grading of meniscal injuries. The clinical surgical validation phase introduces a novel subtype classification.

The aforementioned research substantiates the pivotal role of automated diagnosis and grading in knee meniscal injuries within MRI-based diagnosis of knee joint disorders. These advancements hold promise in providing clinicians with more accurate and consistently timely assessment outcomes. A fine intelligent grading method (VIFG) for meniscal injury is proposed in this study. As shown in Fig. 1, this method mainly consists of three parts, including the automatic segmentation and preprocessing phase, quantitative analysis of injury signal intensity and automatic fine grading stage, and subdivision of subtypes and clinical surgical validation phase. In the clinical surgical validation phase, the anatomical considerations regarding meniscal blood supply were correlated with attention heat maps derived from attributive attention mechanisms. This correlation aimed to complement a more nuanced delineation of secondary injury subtypes. The blood supply status of meniscal injury can also be obtained by MR imaging features alone without the use of invasive arthroscopy, which provides guidance for clinical treatment decisions. The main contributions of this paper can be summarized as follows:

A method (VIFG) was proposed for fine grading of meniscal injury. The attributional attention mechanism accurately and comprehensively localizes injury signals while transfer learning repetitively extracts distinctive global and local features, thereby enhancing the diagnostic efficacy and precision in identifying meniscus injuries. Notably, the grading accuracy on two large MRI datasets is 0.8631 and 0.8502, serving as a foundational basis for precise clinical diagnosis.

The diffusion signal of meniscus injury was characterized by class-activation heat-maps generated by calculating the degree of contribution to the grading. Employing distinct color representations elucidated the progression of diffused signals, challenging for human perception. This approach facilitated the quantification of nuanced intra-meniscal injury traits, significantly enhancing the interpretability of automated fine grading methodologies for meniscal injuries.

Based on the information of the meniscus core and potential injury area shown by the attention heat-map, from the perspective of meniscal blood supply, a refinement of Grade 2 injuries into subtypes 2a, 2b and 2c is proposed, which serves as a preoperative guide for delineating the extent of meniscal resection and has been corroborated through arthroscopic surgical validation.

## Results

### Performance evaluation metrics description

The performance evaluation metrics encompass various indicators. Mean accuracy serves as a comprehensive measure of overall grading accuracy, while Flops and Params quantify the resources allocated by the methods. Within the correlation evaluation metrics, Cohen’s-*κ* correlation coefficient^22^, Pearson’s *r* correlation coefficient^23^, Matthews correlation coefficient^24^ and Jaccard similarity coefficierent^25^ elucidate the correlations between the predicted grades and the ground-truth grades. A coefficient value closer to 1 indicates a stronger correlation. The grading evaluation metrics are defined as follows:

1 $${\rm {Accuracy}}=\frac{{\rm {TP+TN}}}{{\rm {TP+TN+FP+FN}}}$$

2 $${\rm {Specificity}}=\frac{{\rm {TN}}}{{\rm {TN+FP}}}$$

3 $${\rm {Sensitivity}}=\frac{{\rm {TP}}}{{\rm {TP+FN}}}$$

4 $${\rm {Precision}}=\frac{{\rm {TP}}}{{\rm {TP+FP}}}$$

5 $$F1-{\rm {score}}=\frac{2\,{\rm {Sensitivity}}\times {\rm {Precision}}}{{\rm {Sensitivity}}+{\rm {Precision}}}$$

where TP and TN denote the number of correctly classified positive and negative samples, respectively. FP and FN represent the number of misclassified positive and negative samples, respectively. Specifically, the positive samples denote a particular grade, while negative samples encompass the remaining three grades. Accuracy is defined as the ratio of the number of correctly classified samples to the total number of samples. Specificity means the ratio of the number of correctly classified negative samples to the total number of true negative samples. Sensitivity represents the ratio of the number of correctly classified positive samples to the total number of true positive samples. Precision, defined as the ratio of correctly classified positive samples to the total positive samples, provides insight into the classification’s exactness. F1-score is the summed average of precision and sensitivity.

### Meniscal region extraction

**Fig. 2:**
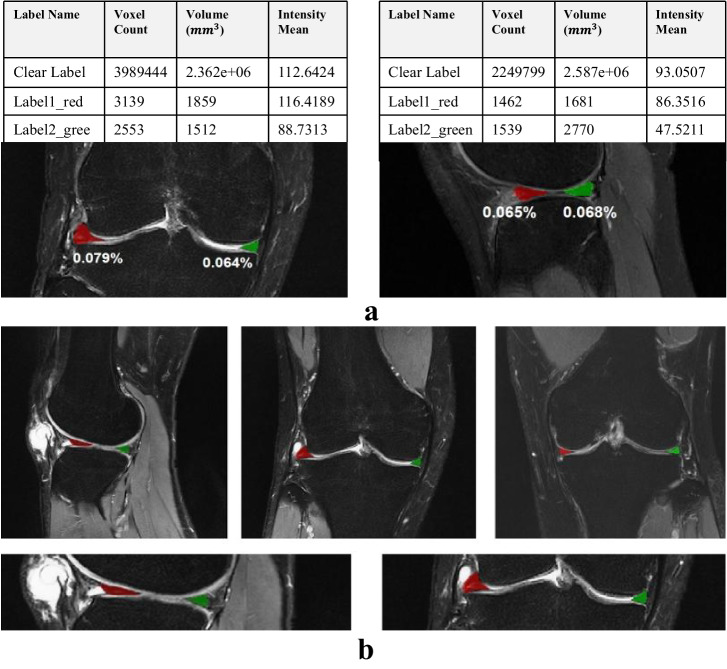
Sketch of meniscus region extraction. **a** Calculated that the meniscus accounted for <0.1 percent of the total knee MRI image. **b** The schematic diagram showed the results of meniscus segmentation in sagittal and coronal MRI.

The meniscus represents a small fraction of the overall knee MRI image, posing a substantial challenge for precise injury grading. As shown in Fig. 2a, the meniscus volume within the MRI scan of the entire knee joint constitutes less than 0.1%. The approach adopted in this study involves segmenting the meniscal region from the entirety of the knee MRI. Leveraging the current optimal segmentation algorithm, a model specializing in meniscus region segmentation was trained. To accommodate variations in data sources and perspectives, four adaptive models were developed for localizing the meniscus region. The average dice similarity coefficient for segmentation was 0.88, as depicted in Fig. 2b showcasing segmentation results. Visualization of the outcomes revealed precise segmentation of the meniscus in both sagittal and coronal orientations. The distinction between two distinct menisci was highlighted using red and green labels. Subsequently, the delineated meniscus region underwent cropping by applying the segmented mask to the original image, creating a refined dataset solely encompassing the meniscal region.

### Meniscus region signal intensity analysis

**Fig. 3:**
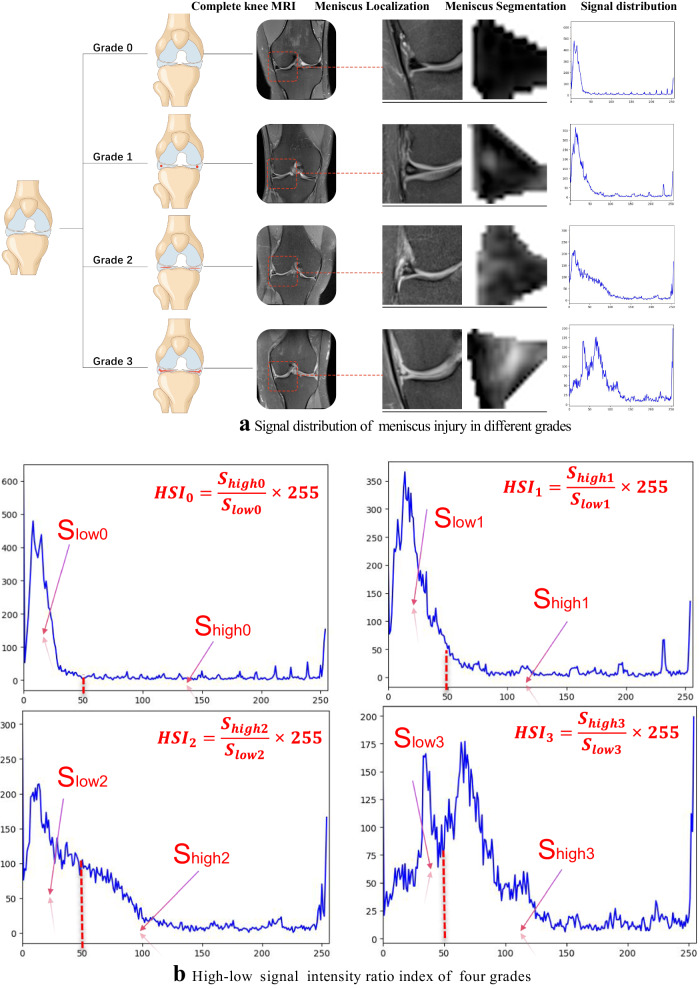
Schematic diagram of meniscus internal injury signal analysis. **a** Represents the signal distribution of meniscus injury of different degrees, and **b** refers to the calculation of the high–low signal intensity ratio index of four grades.

**Fig. 4:**
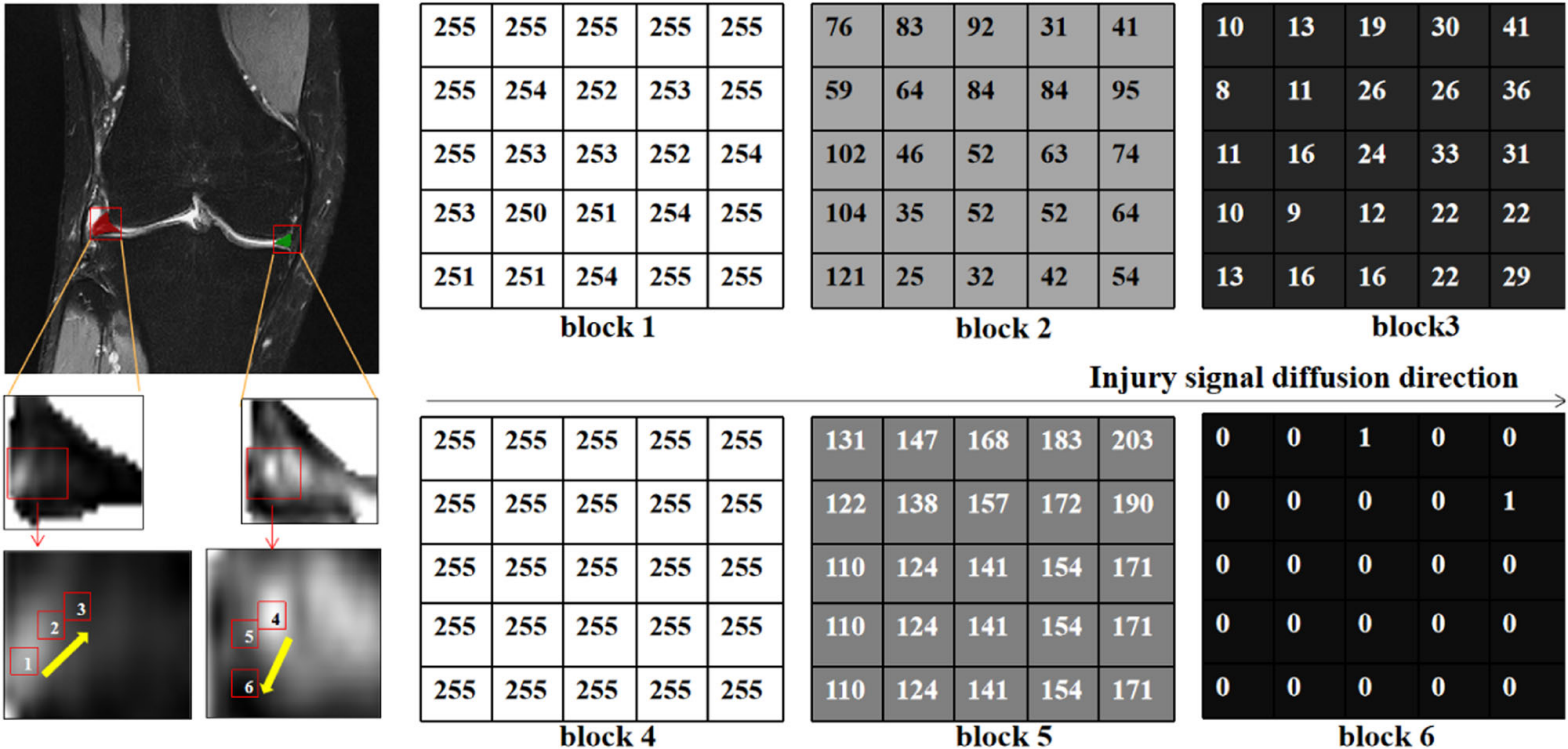
Diagram of the process of gradual change in meniscal injury signal value. Block 1– block 6 are taken from 5*5 pixel blocks in the outward extension direction of the core damage in the meniscus region, respectively.

In clinical settings, T2WI high signal refers to regions with significantly bright signal intensity on T2-weighted imaging. These high signal areas are typically associated with water or fluids, presenting as uniformly white or gray-white on MRI images. In bone MRI imaging, T2WI high signal areas may be related to bone marrow edema, fractures, cartilage lesions, or arthritis, among others. The objective of this study is to grade the severity of meniscal injury through T2WI of the knee joint. We utilized the Fisher grading criteria, where different grades exhibit distinct radiological presentations, as depicted in the schematic diagram in Fig. 3a. Specifically, Grade 0 exhibits a minimal high signal, Grade 1 shows a laminar or circular high signal, Grade 2 displays a horizontal or oblique striped high signal, and Grade 3 demonstrates a high signal extending to the joint surface edge in the meniscus inner fat suppression sequence. Qualitative descriptions based on diagnostic criteria indicate that the focus of meniscal injury grading research lies in the high signal within the meniscus, encompassing the morphology, distribution, and location of high signal, all of which are crucial for injury grading. Therefore, in order to quantitatively define the grading criteria, our study proposed metrics for quantifying the signal within the meniscus. We defined the High-to-Low Signal Intensity Ratio Index (HSI) (as illustrated in Fig. 3) and the signal variation from the injury core to the normal tissue area (as shown in Fig. 4).

Specifically, we introduced the High-to-Low Signal Intensity Ratio Index to assess the severity of meniscal injuries. As depicted in Fig. 3a, in grayscale images, pixel values range from 0 to 255, representing various grayscale levels; a pixel value of 0 signifies pure black, while a value of 255 denotes pure white. Through the output of four signal distribution grades, we observed significant differences when the threshold was set at 50. Consequently, based on the signal intensity distribution within the meniscus images, regions with pixel values >50 were defined as high signal areas (assigned as ${S}_{{\rm {high}}}$), while those below 50 were designated as low signal areas (assigned as ${S}_{{\rm {low}}}$). We then calculated the ratio between these areas and multiplied the result by 255, as shown in the following equation:

6 $${\rm {HS{I}}}_{i}=\frac{{S}_{{\rm {high}}}}{{S}_{{\rm {low}}}}\times 255,i=0,1,2,3$$

The signal intensities for the four grades are denoted as ${\rm {HS{I}}}_{0 - 3}$, distinguishing the four levels as illustrated in Fig. 3b. Through statistical analysis, it was observed that HSI demonstrates an increasing trend with ascending grades. This finding further supplements the existing qualitative grading criteria for meniscal MRI, providing a more quantitative representation of high-signal injury.

Besides quantitatively assessing HSI, signal diffusion analysis was conducted to evaluate the extent of injury influence, depicted in Fig. 4. According to the qualitative grading criteria, the image coloration delineates normal tissue as pure black, the injury core as bright white, and the outward extension of the injured area gradually transitioning from white to black. In examining the signal changes within the injured region, signal values from the injury core and its surrounding blocks were assessed. Remarkably, the injured core area exhibited a conspicuous high signal, with values approaching 255. Moving outward, the injury signal progressively weakened until it became imperceptible to the naked eye. Quantitative output values underscore the evident presence of a core injury and an extended surrounding injury area within the affected meniscus. This progressive pathological process aligns with typical characteristics associated with meniscus injuries.

### Grading results on the different datasets

Before transitioning to more complex methods such as CNNs, we augmented our analysis with machine learning based on radiomics. Utilizing a feature extraction package, we extracted radiomic features from the segmented regions of the meniscus (obtaining 378-dimensional features using the pyradiomics package). Subsequently, we conducted classification experiments using several common machine learning algorithms, including Random Forest, Decision Tree, Naive Bayes, and SVM, thereby introducing additional comparative algorithms in the study. The methods of radiomics can achieve classification tasks in meniscal injury grading. The experimental outcomes, as depicted in Table 2, reveal that the machine learning approach based on radiomics exhibits slightly inferior performance in meniscus injury grading compared to deep learning methods. Due to their excessive reliance on superficial features and the lack of generalizability to large datasets and deep learning is proposed to help solve the problem of deep feature extraction of a large number of data, so this paper adopts the method of deep learning. Substantial experiments confirm the significant advantage of deep learning methods in this task.

**Table 2:** Comparative experimental results of meniscus injury grading with deep learning methods and based on radiology machine learning methods

Method	Accuracy	Precision	Recall	Specificity
*Radiomics-based machine learning*				
Random forest	0.75	0.50	0.68	0.67
Decision tree	0.65	0.65	0.46	0.62
Naive Bayes	0.53	0.49	0.78	0.33
SVM	0.56	0.52	0.73	0.58
*Deep learning*				
ResNet18	0.8343	0.8225	0.8250	0.9449
EfficientNet-B0	0.8429	0.8250	0.8250	0.9482
Swin-transformer	0.8573	0.8224	0.8168	0.9529
Our method	0.8631	0.8550	0.8575	0.9546

**Fig. 5:**
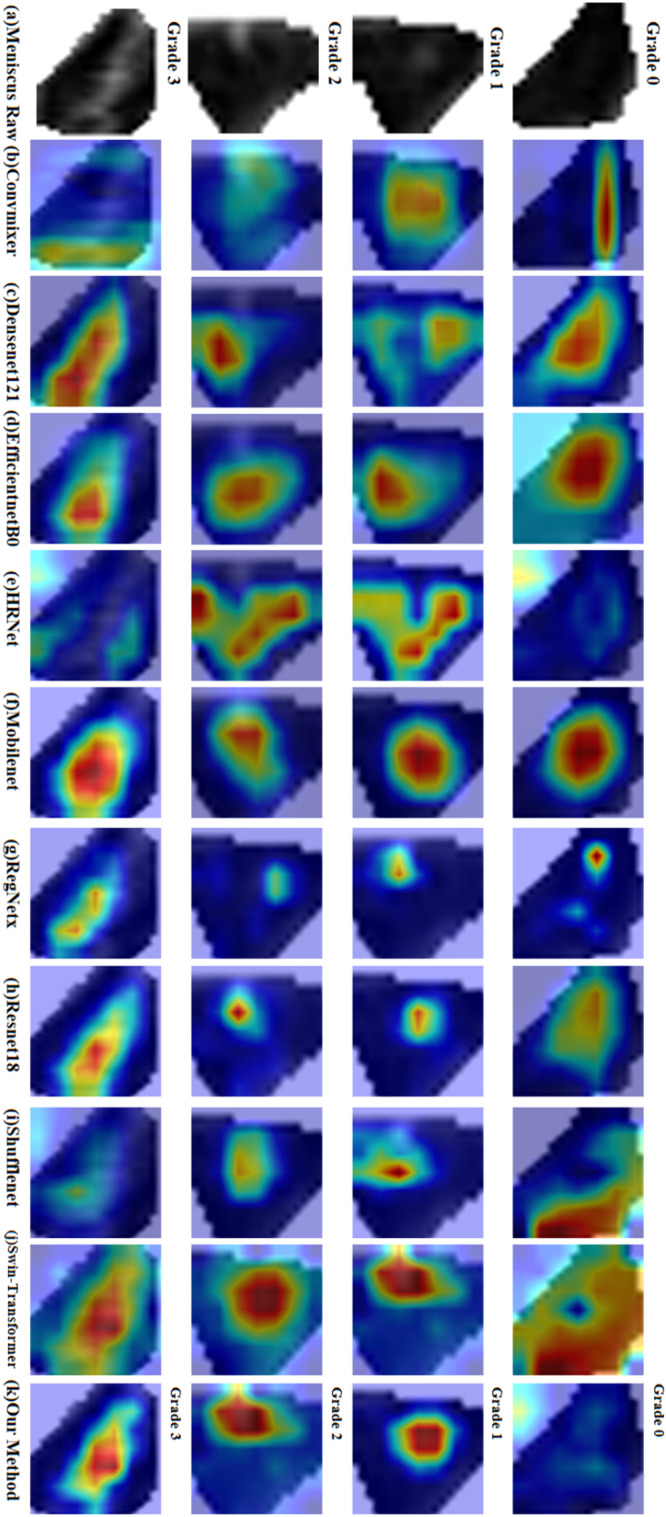
Comparison of visualization results of meniscus grading applying different methods on Xijing_Knee Dataset. **a** Original meniscal region, **b** Convmixer, **c** Densenet, **d** Efficientnet, **e** HRnet, **f** Mobilenet, **g** Regenetx, **h** Resnet18, **i** shufflenet, **j** swin-transformer, and **k** our method.

**Fig. 6:**
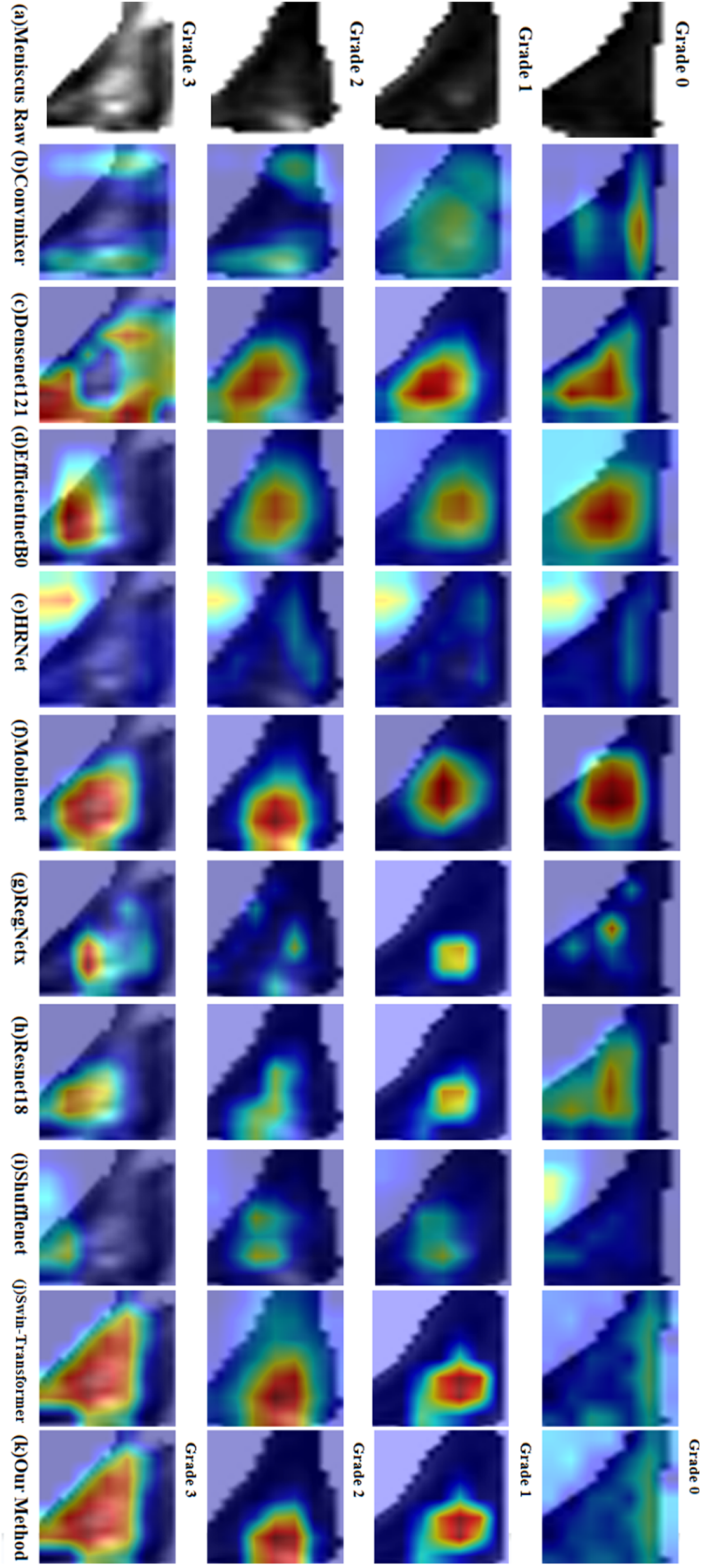
Comparison of visualization results of meniscus grading applying different methods on Xijing_Knee Dataset. **a** original meniscal region, **b** Convmixer, **c** Densenet, **d** Efficientnet, **e** HRnet, **f** Mobilenet, **g** Regenetx, **h** Resnet18, **i** shufflenet, **j** swin-transformer, and **k** our method.

The results obtained by the deep learning method proposed in this paper on the two datasets are shown in Tables 3–6. The visualization results of heat-maps on the two datasets are presented in Fig. 5 and Fig. 6. For the Xijing_Knee dataset, our method achieved a mean accuracy of 85.02% in overall grading performance. Cohen’s kappa correlation coefficient was 0.7982, Pearson’s correlation coefficient was 0.9329, Matthews correlation coefficient was 0.7996, and Jaccard’s similarity coefficient was 0.7478. These quantitative indicators are all superior to other deep learning methods. For the FastMRI_Knee dataset, the mean accuracy of our method in the overall grading performance is 86.31%. Cohen’s kappa correlation coefficient is 0.8258, Pearson’s correlation coefficient is 0.9425, Matthews’s correlation coefficient is 0.8159, and Jaccard’s similarity coefficient is 0.7663. These quantitative indicators are also better compared to other deep learning methods for contrast experiments, as well as better than the Xijing_Knee dataset.

**Table 3:** Comparison results between our method and other deep learning methods on Xijing_Knee dataset

Method	Mean-Acc	Flops	Params	Cohen’s *κ*	JSC	Pearson’s *r*	specificity	MCC	*P*-value
VGG16	0.6123	15.5	138.36M	0.4735	0.5215	0.8573	0.8830	0.4944	0.0061**
DenseNet121	0.7797	2.88	6.96M	0.7029	0.6489	0.8925	0.9267	0.7034	0.0029**
HRNet	0.7974	4.33	19.25M	0.7263	0.6747	0.9160	0.9322	0.7271	0.0057**
RegNetx	0.8282	0.41	4.77M	0.7683	0.7165	0.9288	0.9429	0.7687	0.0088**
ShuffleNet	0.8216	0.15	908.57k	0.7583	0.7145	0.9236	0.9421	0.7617	0.0061**
Convmixer	0.7974	5.55	23.36M	0.7262	0.6785	0.9160	0.9329	0.7281	0.0057**
ResNet101	0.8128	7.85	42.51M	0.7475	0.6963	0.9214	0.9377	0.7479	0.0060**
MobileNet	0.7797	0.32	2.23M	0.7025	0.6622	0.9106	0.9106	0.7116	0.0026**
ResNet152	0.8106	11.58	58.15M	0.7447	0.6903	0.9213	0.9365	0.7450	0.0064**
ResNet18	0.8260	1.82	11.18M	0.7654	0.7128	0.9276	0.9419	0.7657	0.0079**
EfficientNet-B0	0.8150	0.02	4.01M	0.7498	0.7026	0.9214	0.9390	0.7511	0.0058**
Swin-Transformer	0.8260	4.36	27.52M	0.7656	0.7151	0.9302	0.9428	0.7689	0.0105*
Our method	0.8502	3.52	15.26M	0.7982	0.7478	0.9329	0.9502	0.7996	

*Indicates that *P*-value is < 0.05, which is statistically significant; **indicates that *P-*value < 0.001 is statistically significant.

**Table 4:** Comparison of the specific results of four grades on Xijing_Knee dataset

Method	Grade 0				Grade 1				Grade 2				Grade 3			
	Pre	Spe	Rec	F1	Pre	Spe	Recl	F1	Pre	Spe	Rec	F1	Pre	Spe	Rec	F1
VGG16	0.92	0.96	0.56	0.70	0.34	0.75	0.49	0.40	0.14	0.83	0.63	0.23	0.94	0.98	0.75	0.83
DenseNet	0.81	0.93	0.82	0.81	0.75	0.89	0.72	0.74	0.60	0.91	0.68	0.64	0.90	0.96	0.85	0.87
HRNet	0.85	0.94	0.83	0.84	0.76	0.89	0.70	0.73	0.61	0.91	0.73	0.66	0.92	0.97	0.92	0.92
RegNetx	0.90	0.96	0.84	0.87	0.79	0.91	0.77	0.78	0.66	0.92	0.75	0.70	0.92	0.97	0.92	0.92
ShuffleNet	0.94	0.97	0.80	0.86	0.78	0.90	0.74	0.76	0.55	0.90	0.85	0.67	0.93	0.97	0.91	0.92
Convmixer	0.92	0.96	0.78	0.84	0.73	0.88	0.71	0.71	0.58	0.90	0.74	0.65	0.91	0.97	0.95	0.93
ResNet101	0.90	0.96	0.84	0.87	0.78	0.90	0.74	0.76	0.62	0.91	0.70	0.66	0.90	0.96	0.92	0.91
MobileNet	0.98	0.99	0.68	0.81	0.60	0.84	0.71	0.65	0.56	0.90	0.84	0.67	0.93	0.97	0.94	0.93
ResNet152	0.88	0.95	0.84	0.86	0.77	0.90	0.73	0.75	0.65	0.92	0.70	0.68	0.89	0.96	0.94	0.92
ResNet18	0.89	0.96	0.84	0.86	0.78	0.90	0.76	0.77	0.67	0.92	0.75	0.70	0.92	0.97	0.93	0.92
EfficientNet	0.89	0.96	0.84	0.86	0.80	0.91	0.74	0.76	0.57	0.90	0.74	0.64	0.93	0.97	0.91	0.92
Swin-T	0.97	0.99	0.77	0.86	0.70	0.87	0.76	0.73	0.70	0.93	0.80	0.75	0.90	0.96	0.99	0.94
Ours	0.96	0.98	0.82	0.88	0.76	0.90	0.79	0.77	0.74	0.94	0.83	0.78	0.92	0.97	0.98	0.95

Pre represents precision, Spe represents specificity, Rec represents recall, and F1 represents F1-score.

**Table 5:** Comparison results between our method and other deep learning methods on FastMRI_Knee dataset

Method	Mean-Acc	Flops	Params	Cohen’s *κ*	JSC	Pearson’s *r*	specificity	MCC	*P*-value
VGG16	0.6282	15.5	138.3M	0.4912	0.5434	0.8601	0.8877	0.5108	0.0057**
DenseNet121	0.7810	2.88	6.96M	0.7065	0.6443	0.9033	0.9261	0.7082	0.0038**
HRNet	0.8285	4.33	19.25M	0.7698	0.7143	0.9314	0.9427	0.7701	0.0060**
RegNetx	0.8401	0.41	4.77M	0.7846	0.7313	0.9339	0 .9469	0.7848	0.0055**
ShuffleNet	0.8343	0.15	908.5k	0.7761	0.7308	0.9376	0.9459	0.7775	0.0090**
Convmixer	0.8386	5.55	23.36M	0.7826	0.7309	0.9369	0.9470	0.7837	0.0081**
ResNet101	0.8386	7.85	42.51M	0.7825	0.7344	0.9346	0.9468	0.7829	0.0057**
MobileNet	0.8098	0.32	2.23M	0.7428	0.7025	0.9170	0.9391	0.7449	0.0039**
ResNet152	0.8501	11.5	58.15M	0.7982	0.7488	0.9401	0.9505	0.7985	0.0086**
ResNet18	0.8343	1.82	11.18M	0.7769	0.7259	0.9361	0.9449	0.7773	0.0075**
EfficientNet-B0	0.8429	0.02	4.01M	0.7884	0.7391	0.9403	0.9482	0.7887	0.0106*
Swin-Transformer	0.8573	4.36	27.52M	0.8082	0.7595	0.9442	0.9529	0.8089	0.0305*
Our method	0.8631	3.52	15.26M	0.81581	0.7663	0.9425	0.9546	0.8159	

*Indicates that *P-*value is < 0.05, which is statistically significant; **indicates that *P*-value < 0.001 is statistically significant.

**Table 6:** Comparison of the specific results of four grades on FastMRI_Knee dataset

Method	Grade 0				Grade 1				Grade 2				Grade 3			
	Pre	Spe	Rec	F1	Pre	Spe	Rec	F1	Pre	Spe	Rec	F1	Pre	Spe	Rec	F1
VGG16	0.89	0.94	0.62	0.73	0.38	0.79	0.46	0.42	0.12	0.82	0.81	0.21	0.98	0.99	0.73	0.84
DenseNet	0.79	0.91	0.91	0.84	0.73	0.90	0.64	0.68	0.71	0.92	0.65	0.68	0.87	0.96	0.93	0.90
HRNet	0.89	0.95	0.94	0.91	0.78	0.91	0.91	0.76	0.71	0.92	0.68	0.68	0.88	0.96	0.92	0.90
RegNetx	0.91	0.96	0.88	0.90	0.78	0.92	0.75	0.77	0.72	0.93	0.76	0.74	0.89	0.96	0.93	0.91
ShuffleNet	0.95	0.97	0.87	0.91	0.77	0.91	0.71	0.74	0.63	0.91	0.77	0.70	0.92	0.97	0.96	0.94
Convmixer	0.95	0.97	0.85	0.89	0.72	0.90	0.78	0.75	0.76	0.94	0.75	0.76	0.88	0.96	0.96	0.92
ResNet101	0.94	0.97	0.87	0.90	0.73	0.90	0.74	0.74	0.71	0.92	0.76	0.73	0.73	0.97	0.95	0.94
MobileNet	0.96	0.98	0.84	0.89	0.72	0.90	0.73	0.73	0.55	0.89	0.73	0.63	0.92	0.97	0.89	0.90
ResNet152	0.94	0.97	0.88	0.91	0.76	0.91	0.77	0.76	0.74	0.93	0.76	0.75	0.92	0.97	0.96	0.94
ResNet18	0.91	0.96	0.91	0.91	0.78	0.91	0.72	0.75	0.68	0.92	0.73	0.71	0.92	0.97	0.94	0.93
EfficientNet	0.93	0.97	0.90	0.92	0.79	0.92	0.75	0.77	0.68	0.92	0.74	0.71	0.90	0.97	0.94	0.92
Swin-T	0.94	0.97	0.88	0.91	0.72	0.90	0.80	0.76	0.81	0.95	0.76	0.78	0.93	0.97	0.95	0.94
Ours	0.93	0.97	0.92	0.92	0.81	0.93	0.78	0.80	0.75	0.94	0.79	0.77	0.93	0.98	0.94	0.93

Pre represents precision, Spe represents specificity, Rec represents recall, and F1 represents F1-score.

The results of statistical significance tests were presented in Tables 3 and 5 to ascertain the significance of the outcomes. Paired *t*-tests were employed, and additional experiments were conducted to compute the t-statistic and the corresponding *p*-value. Throughout the computation process, our method served as the baseline against which the results of other methods were compared for statistical significance. The obtained results are displayed in the last column of Tables 3 and 5. The *p*-values indicate that at a significance level of 0.05, our method exhibits superior performance compared to other CNN architectures, with statistically significant differences noted as *p < 0.05 and **p < 0.01.

To measure the performance of our method on the four grades respectively, precision, specificity, recall, and F1-score were calculated on the four grades. The experimental results show that all deep learning methods can perform well in Grades 0 and 3. The superiority of our approach is mainly reflected in Grade 1 and Grade 2, which are the two most difficult grades to distinguish. The details are as follows, for the Xijing_Knee dataset, Grade 1’s precision is 0.763, specificity is 0.9, recall is 0.786, and F1-score is 0.774. Grade 2’s precision is 0.741, specificity is 0.941, specificity is 0.829, and F1-score 0.783. For the FastMRI_Knee dataset, Grade 1 has a precision of 0.810, specificity of 0.930, recall of 0.784, and F1-score is 0.797. Grade 2 has a precision of 0.754, specificity of 0.939, recall of 0.794, and F1-score is 0.773. Our method performed better on grading quantitative indicators of Grade 1 and Grade 1.

Due to the sufficient quantity of both datasets, five-fold cross-validation was carried out on the private dataset XijingMRI_Knee and the public dataset Fast MRI_Knee, respectively, and the results obtained were shown in Tables 7 and 8. Our method exhibits outstanding performance in the five-fold cross-validation experiments, displaying evident advantages compared to other typical methods. Particularly noteworthy is the attainment of the highest accuracy of 89.33% in the second-fold experiment on the XijingMRI_Knee dataset and an accuracy of 87.54% in the fourth-fold experiment on the FastMRI_Knee dataset. From the average classification accuracy of the five-fold cross-validation experiments, it can also be observed that our method demonstrates a certain level of stability.

**Table 7:** The results of the five-fold cross-validation experiment on the XijingMRI_Knee dataset, with accuracy as the index

Method|	Fold 1	Fold 2	Fold 3	Fold 4	Fold 5	Average
VGG16	0.3267	0.2867	0.5267	0.6267	0.6689	0.4871
DenseNet	0.8160	0.7864	0.7537	0.7507	0.7033	0.7620
HRNet	0.7133	0.8067	0.7867	0.7733	0.7483	0.7657
RegNetx	0.7933	0.8443	0.7800	0.8133	0.8079	0.8078
ShuffleNet	0.7820	0.8100	0.7963	0.8037	0.8163	0.8017
Convmixer	0.7774	0.7270	0.7685	0.7982	0.6558	0.7454
ResNet101	0.8200	0.7800	0.8200	0.8200	0.7947	0.8069
MobileNet	0.7933	0.8333	0.7867	0.7267	0.7947	0.7869
ResNet152	0.8400	0.7467	0.8400	0.8200	0.7947	0.8083
ResNet18	0.7800	0.8523	0.7600	0.7933	0.8278	0.8027
EfficientNet	0.7933	0.8367	0.7867	0.8200	0.8575	0.8188
Swin-T	0.8576	0.8200	0.7948	0.8267	0.8133	0.8245
Ours	0.8200	0.8933	0.8309	0.8600	0.8423	0.8493

**Table 8:** The results of the five-fold cross-validation experiment on the FastMRI_Knee dataset, with accuracy as the index

Method|	Fold 1	Fold 2	Fold 3	Fold 4	Fold 5	Average
VGG16	0.7151	0.4214	0.6825	0.6231	0.6558	0.6196
DenseNet	0.7537	0.7418	0.8131	0.7745	0.7626	0.7691
HRNet	0.8338	0.8101	0.8487	0.8101	0.7982	0.8202
RegNetx	0.8427	0.8190	0.8309	0.8487	0.7893	0.8261
ShuffleNet	0.8220	0.8220	0.8427	0.8071	0.7834	0.8154
Convmixer	0.7567	0.7567	0.7211	0.7151	0.7181	0.7335
ResNet101	0.8398	0.7834	0.8042	0.8071	0.7893	0.8048
MobileNet	0.8249	0.8160	0.8131	0.7715	0.8190	0.8089
ResNet152	0.7982	0.8190	0.8042	0.8665	0.8220	0.8220
ResNet18	0.8398	0.8427	0.8071	0.7953	0.8220	0.8214
EfficientNet	0.8427	0.8427	0.8131	0.8338	0.7982	0.8261
Swin-T	0.7953	0.8012	0.8576	0.8190	0.8398	0.8226
Ours	0.8427	0.8279	0.8605	0.8754	0.8665	0.8546

Regarding the external testing perspective, the external testing was performed by utilizing the test segment of the private dataset Xijing_Knee as the external test for the model trained on the public dataset Fast MRI_Knee. The outcomes of the external testing, as depicted in Tables 9 and 10, validate the robustness of the algorithm. Comparative analysis across Mean-Acc, Cohen’s *κ*, JSC, Pearson’s *r*, specificity, MCC, and specific grading metrics at each level demonstrates superior performance of our method in independent testing results compared to other methods. The best Mean-Acc reaches 92.5%, providing substantial evidence for the robustness of this approach than the comparison algorithm and baseline algorithm on two datasets.

**Table 9:** Cross-center data independent test results

Method	Mean-Acc	Cohen’s *κ*	JSC	Pearson’s *r*	specificity	MCC	*p*-value
VGG16	0.6150	0.4764	0.5250	0.8583	0.8839	0.4978	0.0052**
DenseNet121	0.7780	0.7004	0.6380	0.8992	0.9250	0.7019	0.0044**
HRNet	0.8550	0.8043	0.7511	0.9423	0.9513	0.8046	0.0063**
RegNetx	0.8630	0.8158	0.7656	0.9427	0.9545	0.8164	0.0056**
ShuffleNet	0.8770	0.8344	0.7836	0.9507	0.9586	0.8356	0.0069**
Convmixer	0.8550	0.8048	0.7513	0.9456	0.9517	0.8076	0.0071**
ResNet101	0.8570	0.8070	0.7565	0.9423	0.9523	0.8074	0.0060**
MobileNet	0.9070	0.8754	0.8357	0.9583	0.9695	0.8763	0.0046**
ResNet152	0.8630	0.8159	0.7643	0.9443	0.9544	0.8163	0.0061**
ResNet18	0.8330	0.7743	0.7195	0.9313	0.9437	0.7749	0.0055**
EfficientNet-B0	0.8370	0.7799	0.7314	0.9348	0.9461	0.7808	0.0057**
Swin-Transformer	0.8810	0.8402	0.7931	0.9548	0.9601	0.8409	0.0076**
Our method	0.9250	0.8990	0.8631	0.9664	0.9752	0.8995	

The test set part of the XijingMRI_Knee dataset is tested on the model trained by the FastMRI_Knee dataset.

**Indicates that *P*-value < 0.001 is statistically significant.

**Table 10:** The test set part of Xijing_Knee dataset is tested on the model trained by FastMRI_Knee dataset, and the specific performance of each grade was obtained

Method	Grade 0				Grade 1				Grade 2				Grade 3			
	Pre	Spe	Rec	F1	Pre	Spe	Rec	F1	Pre	Spe	Rec	F1	Pre	Spe	Rec	F1
VGG16	0.92	0.96	0.56	0.70	0.34	0.75	0.49	0.40	0.14	0.83	0.66	0.23	0.95	0.98	0.75	0.84
DenseNet	0.76	0.91	0.85	0.80	0.78	0.90	0.68	0.73	0.69	0.92	0.66	0.67	0.84	0.94	0.92	0.88
HRNet	0.89	0.96	0.92	0.91	0.84	0.93	0.79	0.82	0.74	0.94	0.73	0.73	0.90	0.96	0.95	0.92
RegNetx	0.92	0.97	0.86	0.89	0.84	0.93	0.80	0.82	0.72	0.93	0.80	0.76	0.92	0.97	0.97	0.94
ShuffleNet	0.94	0.97	0.83	0.88	0.79	0.91	0.87	0.82	0.85	0.96	0.82	0.83	0.92	0.97	0.98	0.95
Convmixer	0.95	0.98	0.78	0.86	0.74	0.89	0.84	0.78	0.82	0.95	0.81	0.81	0.90	0.96	1.00	0.95
ResNet101	0.92	0.97	0.87	0.89	0.83	0.92	0.80	0.81	0.71	0.93	0.78	0.74	0.92	0.97	0.96	0.94
MobileNet	0.99	0.99	0.88	0.93	0.85	0.94	0.88	0.87	0.80	0.95	0.88	0.84	0.95	0.98	0.98	0.96
ResNet152	0.90	0.96	0.88	0.89	0.85	0.93	0.80	0.83	0.74	0.94	0.79	0.76	0.91	0.97	0.96	0.93
ResNet18	0.86	0.95	0.87	0.86	0.81	0.91	0.74	0.78	0.70	0.93	0.74	0.72	0.91	0.97	0.97	0.94
EfficientNet	0.91	0.96	0.87	0.89	0.82	0.92	0.76	0.79	0.63	0.91	0.75	0.68	0.92	0.97	0.95	0.93
Swin-T	0.95	0.98	0.85	0.90	0.79	0.91	0.84	0.82	0.83	0.96	0.82	0.83	0.94	0.98	0.99	0.96
Ours	0.96	0.98	0.91	0.93	0.90	0.95	0.88	0.89	0.83	0.96	0.95	0.89	0.97	0.99	0.96	0.97

Pre represents precision, Spe represents specificity, Rec represents recall, and F1 represents F1-score.

In addition to the quantitative results of grading, qualitative visual results were also obtained. Due to the heatmaps’ proficiency in visually representing the locations, shapes, and extents of distinct features indicative of different grades of meniscus injury in imaging, we proposed the integration of an attributional attention module in the refined classification process of meniscus injuries. This module precisely guides the model to focus on regions that contribute significantly to injury grading. The model’s emphasis on high-intensity areas is essential due to the clinical diagnostic standards wherein highlighted information reflects manifestations of tissue fluids, tears, and aligns with the focal points of diagnostic grading criteria. Therefore, it is imperative for the model to attend to these highlighted regions.

Our approach distinctly illustrates discernible differences in visualized heatmaps across various grades. Based on the results of visualizing heat-maps from different methods on the two datasets, it was found that deep learning algorithms of contrast miss critical injury when localizing injured signals. And compared to baseline method, our method can accurately locate the injured location and show the core injured signal with finer focus. As depicted in the additional Fig. 7a–d represent correctly classified samples corresponding to Grade 0 to Grade 3, along with their respective heatmaps. The heatmaps for Grade 0 essentially lack prominently highlighted areas, aligning with diagnostic standards. For Grade 1, the highlighted areas are primarily concentrated within the meniscus, forming circular regions with a relatively smaller range. Grade 2 heatmaps display broader highlighted areas compared to Grade 1, covering larger impact regions, mostly located closer to the joint capsule. Grade 3 heatmaps exhibit the widest range of highlighted areas, occupying nearly half of the meniscus area, spanning across regions near the joint capsule to the articular surface. Each grade’s heatmaps exhibit distinctive characteristics, aligning with clinical standards for meniscus injury grading based on Fisher’s grading criteria, demonstrating the interpretability of heatmaps by vividly presenting the locations, shapes, and extents of injury signals.

**Fig. 7:**
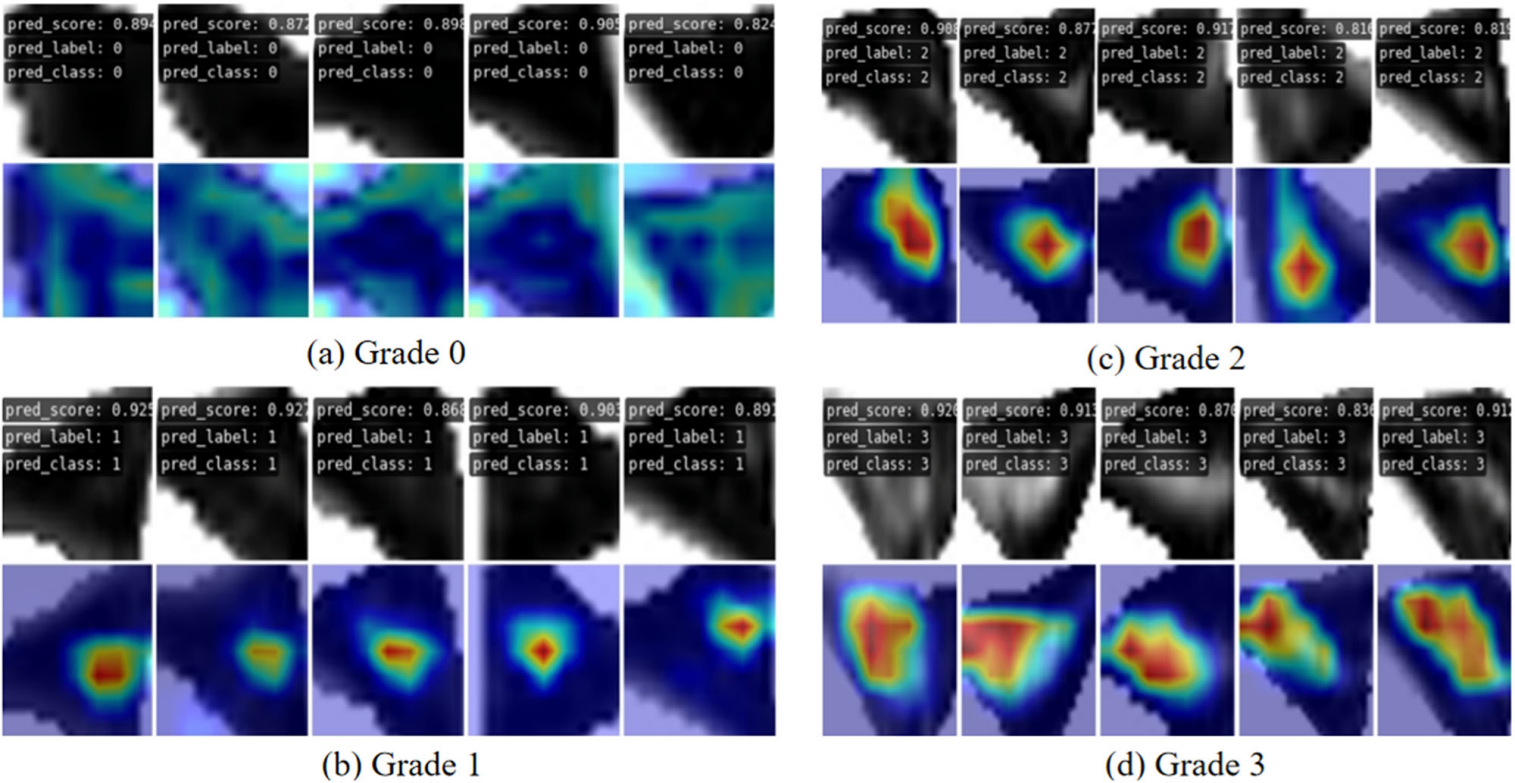
Four grades of correct prediction results and corresponding heat maps. **a** is the prediction result and thermal map corresponding to Grade 0, and it can be seen that no obvious highlighted area is seen. **b**–**d** is the prediction result and thermal map corresponding to Grade 1, Grade 2 and Grade 3, respectively. It can be seen that the area of the highlighted area has increased significantly and the range is wider.

Overall, the FastMRI_Knee datase performed better by comparing the above quantitative and visual results on the two datasets. This is motivated by the two following reasons, the large amount of data and the higher degree of injury signal aggregation. The influence of meniscus injury grading results not only comes from the size of data but also is related to the location of meniscus injury, the degree of diffusion and the degree of signal aggregation. Therefore, our method combines the advantages of large-scale data samples, accurate injury location algorithm and quantitative injury signal analysis to make correct fine-grading diagnosis.

**Fig. 8:**
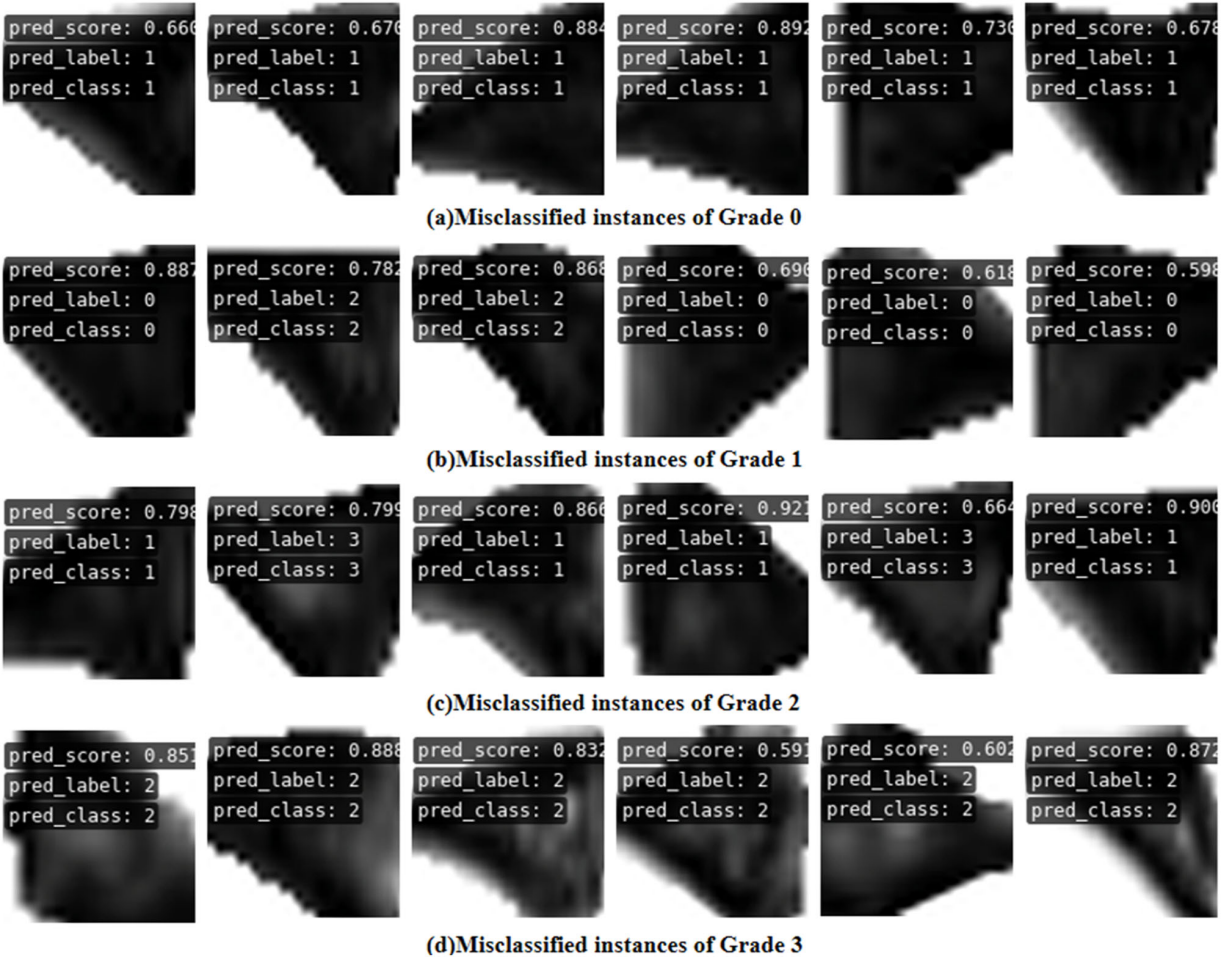
Schematic illustration of the failure cases. **a**–**d** correspond to Grade 1–Grade 3 grading failure cases, where failure cases are mainly misclassified into adjacent injury grades.

**Fig. 9:**
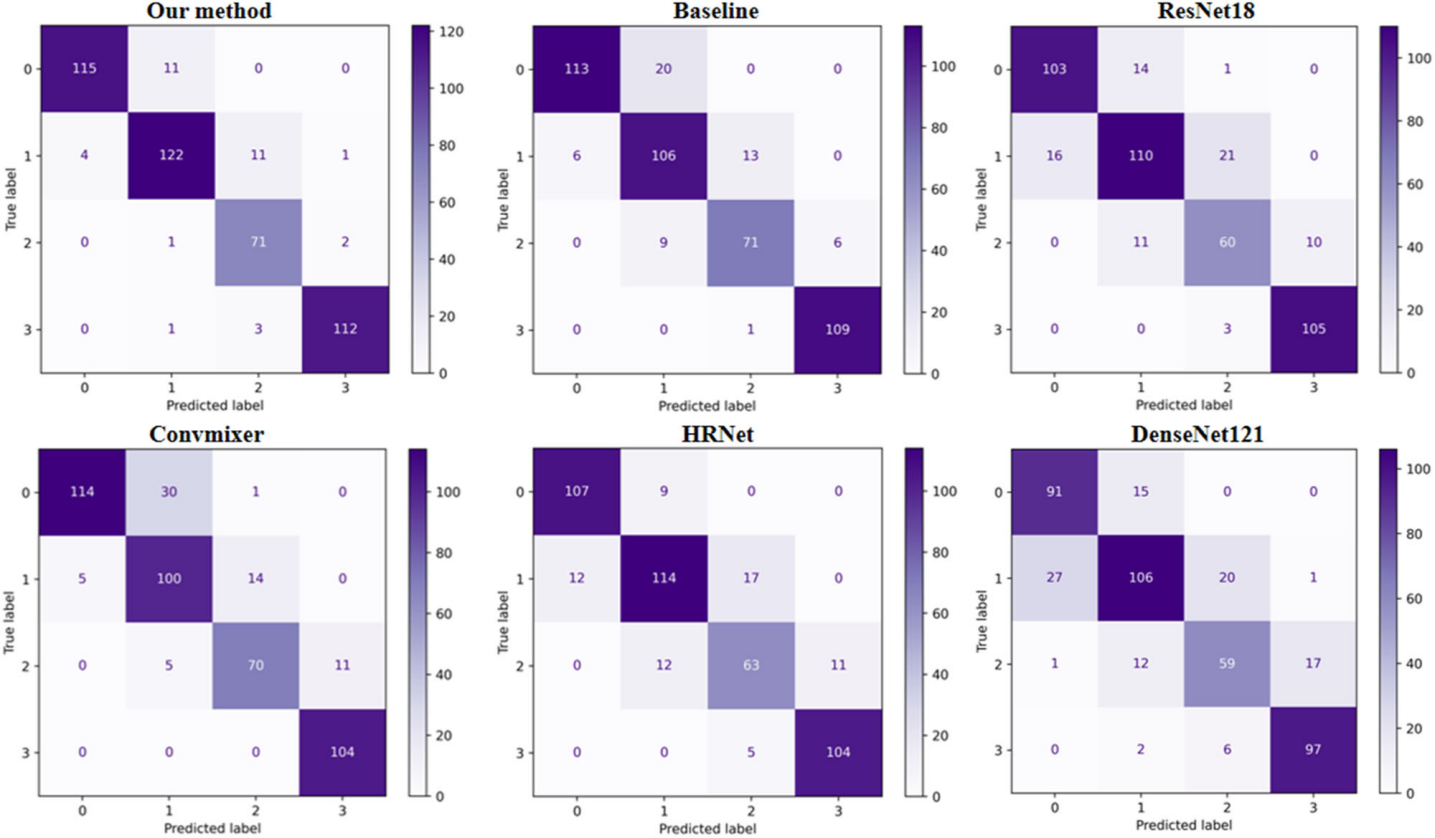
Confusion matrix for classification results on the Xijing_Knee dataset. The confusion matrix consists of six algorithms, respectively, and the first graph is our method, which can be seen to have the best grading performance.

In Fig. 8, instances of prediction errors were reported, with misclassified samples observed across each grade. Notably, Grades 0 and 3 exhibit higher accuracy rates and fewer misclassifications. Misclassifications in Grade 0 predominantly result in predictions as Grade 1, while Grade 3 misclassifications are primarily predicted as Grade Conversely, Grades 1 and 2, which pose greater grading difficulties, are more prone to misclassification as adjacent grades. Further clarification on specific classification scenarios is provided through the illustrative confusion matrices in Fig. 9. Analysis of misclassified data allows for the identification of three primary contributing factors: firstly, issues related to image quality arise due to differences in image acquisition protocols within the dataset, resulting in varying image qualities. Secondly, individual variations in case profiles and subtle features among grades contribute to misclassifications. Thirdly, the method proposed in this paper has some limitations, demonstrating good classification capabilities concerning grade boundaries but encountering challenges in accurately classifying ambiguous cases, particularly Grades 1 and 2, which possess unclear classification boundaries.

### Correlation study of meniscus injury grading

Due to the advantage of a large amount of data in this study, we conducted a correlation study between meniscus injury grade and various influencing factors of subjects to explore the trend of meniscus injury in the population and obtain a more complete and detailed evaluation system. We concluded that age, sex, body weight, medial or lateral meniscus were significantly correlated with Fisher’s grade of meniscus injury, while Fisher’s grade of meniscus injury was not significantly correlated with left and right legs. Non-imaging variables such as gender, age, and weight can be important for this task. We plan to incorporate the experimental findings pertaining to this section. Furthermore, we aim to incorporate non-imaging variables such as gender, age, and weight into the model as non-imaging features embedded within the network, leading to the results shown in Table 11. It is evident that age, gender and weight, as non-imaging features, contribute to an improvement in the grading performance. From the experimental results, the addition of gender and weight separately showed a slight improvement in the grading accuracy. However, after introducing age as a single feature, there was a more noticeable enhancement in grading accuracy. Embedding all three non-imaging features into the network yielded the best grading performance.

**Table 11:** (a) Non-imaging variables such as gender, age, weight are embedded in the algorithm for the overall experimental results on the FastMRI_Knee dataset. (b) The specific experimental results of the embedding of non-imaging variables at each grade were graded on the FastMRI_Knee dataset

(a)								
Method			Mean-Acc	Cohen’s *κ*	JSC	Pearson’s *r*	specificity	MCC
Our method			0.8631	0.81581	0.7663	0.9425	0.9546	0.8159
+Gender	+Weight	+Age						
√			0.8700	0.8241	0.7784	0.9470	0.9575	0.8266
	√		0.8720	0.8275	0.7813	0.9493	0.9579	0.8291
		√	0.9050	0.8723	0.8312	0.9609	0.9683	0.8726
√		√	0.9190	0.8903	0.8510	0.9619	0.9729	0.8909
√	√	√	0.9210	0.8930	0.8588	0.9671	0.9742	0.8936

(b)														
Method			Grade 0			Grade 1			Grade 2			Grade 3		
			Pre	Rec	F1	Pre	Rec	F1	Pre	Rec	F1	Pre	Rec	F1
Our method			0.93	0.92	0.92	0.81	0.78	0.80	0.75	0.79	0.77	0.93	0.94	0.93
+Gender	+Weight	+Age												
√			0.95	0.89	0.92	0.88	0.76	0.82	0.67	0.85	0.75	0.91	1.00	0.95
	√		0.97	0.84	0.90	0.83	0.81	0.82	0.71	0.85	0.78	0.93	0.99	0.96
		√	0.94	0.90	0.92	0.87	0.84	0.85	0.81	0.90	0.85	0.97	0.98	0.97
√		√	0.98	0.89	0.93	0.88	0.90	0.89	0.85	0.88	0.86	0.93	0.99	0.96
√	√	√	0.98	0.95	0.96	0.93	0.86	0.90	0.77	0.86	0.82	0.94	0.99	0.97

Mean-Acc indicates average accuracy, Cohen’s-κ stands for Cohen’s-κ correlation coefficient, JSC indicates Jaccard similarity, Pearson’s r stands for Pearson’s r correlation coefficient and MCC indicates Matthews correlation coefficient. Pre represents precision, Spe represents specificity, Rec represents recall, and F1 represents F1-score.

### Clinical significance of injury heatmaps

**Fig. 10:**
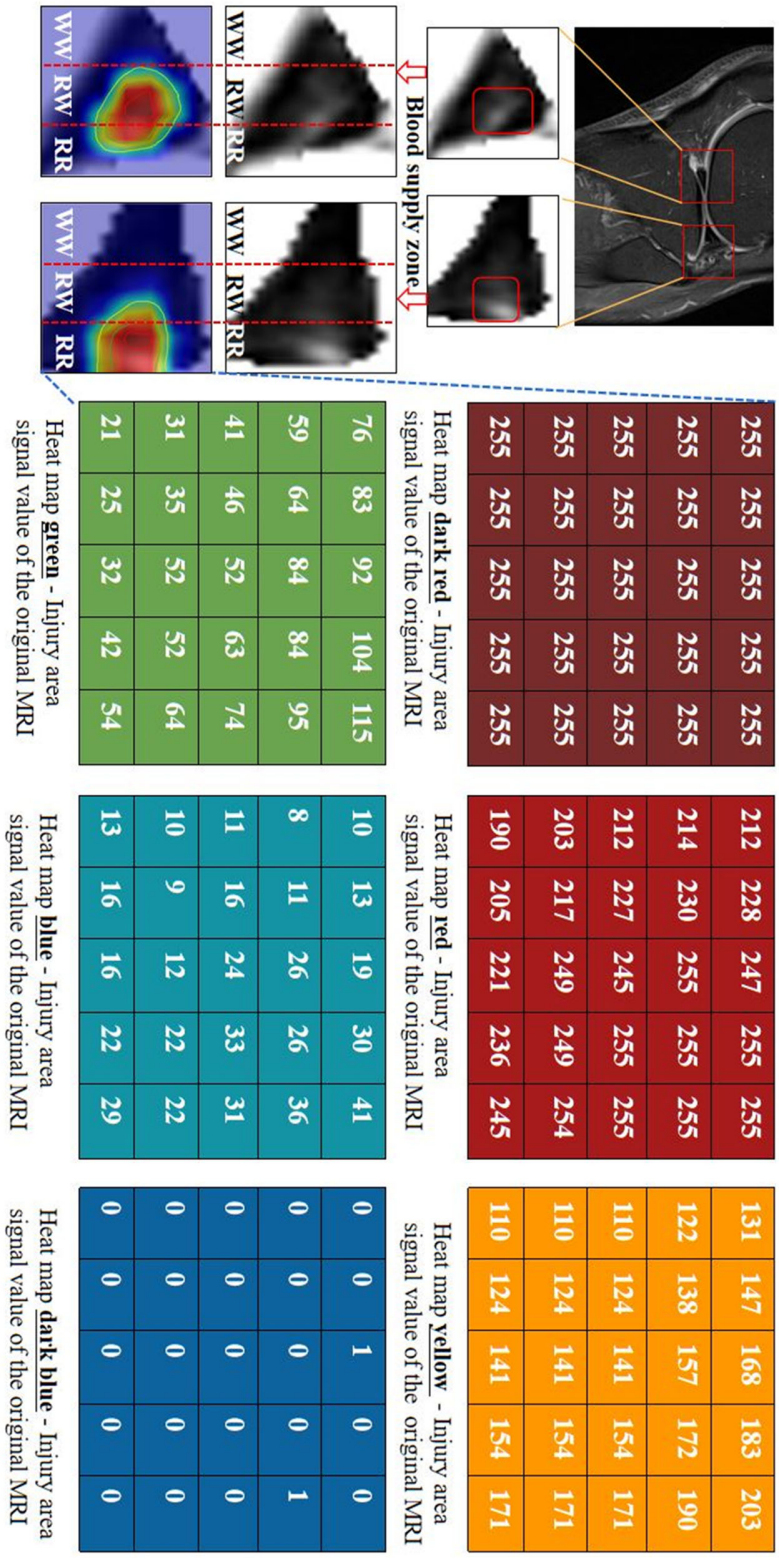
Diagram of injury diffusion. The heat-maps for the location of the injury core and diffusion of the injury changes in the extended area. The sequence of changes in dark red, red, yellow, green, blue and dark blue, respectively, represents the changes in the outward extension direction of the core damage in the meniscus region. The numbers on each color block represent pixel values on the original MRI image of the meniscus.

This research article incorporates the attributional attention module within the VIFG framework, yielding attentional heat maps crucial for precise localization of the affected area. As shown in Fig. 10, the signal blocks of the heat-maps are consistent with the conclusions obtained above section of meniscus region signal intensity analysis. Notably, the core injury region exhibits a prominent high signal, nearing values of 255, while the signal weakens with outward dispersion. These heat maps employ varied colors to denote the severity and spatial extent of the injury. The dark red area is the signal value corresponding to the location of the core of the injury, and the red area represents the signal value referring to the area most adjacent to the location of the core injury. The signal values within the yellow area register lower values compared to the red area, ranging from 100 to 200, all within the range perceptible to the human eye. As the injured area extends into the green area, it is barely discernible to our naked eye, but it shows a difference in signal values from the normal tissue of the meniscus represented by dark blue. On the basis of different color blocks representing different injury degrees, a quantitative evaluation index AIA was obtained by calculating the sum of the areas of core dark red and extended red areas. Hence, our proposed method enables a refined grading analysis of meniscal injuries and non-invasive localization of the affected area, significantly enhancing diagnostic efficiency.

### Subtypes representation and anatomic verification

The proposed method realizes the intelligent grading across the spectrum of the four grades of meniscal injury, utilizing attentional heat maps to pinpoint the site of injury. In clinical scenarios, Grade 2 holds paramount importance in determining the course of clinical treatment. However, experimental findings indicate that Grade 2 poses exceptional difficulty and presents the most formidable challenge across diverse methodologies, emerging as the weakest performer among the four grades. Because surgical planning for knee meniscal injury necessitates consideration not only of the injury type and tear magnitude but also critically relies on the local blood supply at the injury site. To achieve precise therapeutic outcomes and optimize meniscal function preservation in patients, minimal resection or suturing of the injured meniscus is imperative.

**Fig. 11:**
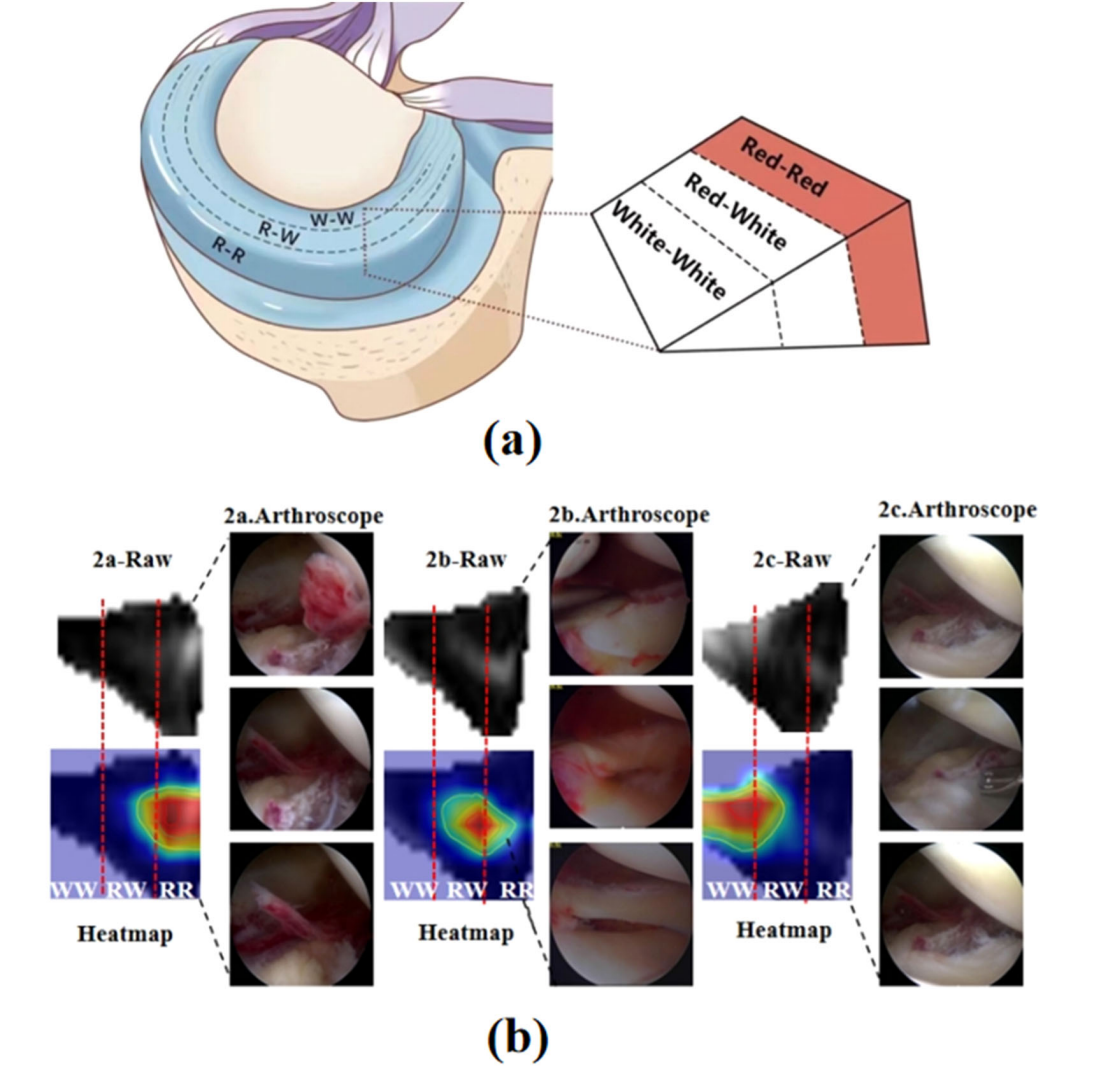
Schematic diagram of subtypes representation and anatomical verification. **a** Meniscus blood supply relation position anatomical diagram. Dividing the meniscus region into three equal parts, the segment proximal to the joint capsule is termed the red-red area (RR), the intermediate area designates the red-white area (RW), and the innermost portion corresponds to the white-white area (WW). **b** The blood supply region corresponding to the heat-maps and the results of arthroscopic verification. The figure showed the original MRI image of the 2a red-red area, 2b red-white area and 2c white area, the thermal map of the injured area and the video capture of arthroscopic surgery, respectively. Clinically, arthroscopy was used to verify the blood supply shown by the heat-maps of attention.2a is the arthroscopic video effect of red and red area 2a, 2b is the arthroscopic observation results of red and white area 2b, and 2c is the arthroscopic operation image of white and white area 2c.

In this paper, based on the research results and combined with the vascular distribution of the meniscus, the anatomical knowledge that the meniscus is divided into three equal parts by distance from the outside to the inside was presented^26^. As shown in Fig. 11 (a), the outer third of the meniscus is often referred to as the “red zone” because it has a good blood supply and is capable of healing on its own if the tear is small and does not necessarily require to be treated surgically. There is a partial blood supply in the middle third of the meniscus, known as the “red-white zone,” and it is less likely to heal on its own. The “white area” in the inner third of the meniscus has little to no blood supply and cannot heal on its own once the injury has occurred, only to be surgically removed. The entire meniscus is evenly divided into three parts according to the whole distance from the capsule to the innermost part of the joint. The first third is red and red areas, indicating an adequate blood supply. The middle third is red and white, representing limited blood supply. The final third of the area is white, representing almost no blood flow. The middle third is red and white, representing limited blood supply. The final third of the area is white, representing almost no blood flow. The heat-maps were employed to delineate the location and extent of the core injury area based on concentrated dark red and red zones. In cases where the injury spans across multiple areas, priority was accorded to the most severe region. To furnish a more nuanced representation of injury severity, subtypes were proposed within Grade 2, specifically 2a (red-red zone), 2b (red-white zone), and 2c (white zone). The meniscus was divided into three equal segments from the inner to outer regions, as illustrated in Fig. 11b. Within this scheme, 2a indicates core injury about one-third of the way up the joint capsule, 2b signifies the lesion’s midsection, and 2c encompasses the remaining distal capsule area. Integrating subtype grading with clinical practice facilitated an understanding of the relationship between heat map-identified regions and anatomical blood supply findings, validated in select clinical scenarios.

The specifics are detailed in Fig. 11b. The left panel delineates the locations of the injured areas corresponding to RR, RW, and WW regions. These position relationships provide the anatomic basis for the classification of subtypes of knee meniscus injuries. This novel subtyping protocol was validated through arthroscopic surgery for meniscus injuries, as depicted in the right panel of Fig. 11b. The heat-maps of 2a show that the core of the injury to be in the RR region. During arthroscopic surgery, the injury core, found near the joint capsule and exhibiting pronounced vascularity, manifested a more hemoglobin-rich coloration. For 2b, the predominant injured core was identified within the RW region on the heat-maps, with some discernible vascularity observed during arthroscopic surgery. Regarding 2c, the core of injury was mainly in the WW area, corroborated by arthroscopic surgery footage revealing an absence of appreciable blood supply. The findings of this study can complement the current qualitative grading system for meniscal injury, providing enhanced guidance for clinical diagnosis and treatment through quantified and visualized outcomes.

## Discussion

The superiority of our method in grading of knee meniscal injury. Our proposed method (VIFG) exhibited remarkable efficacy in fine grading validation using both the public FastMRI_Knee dataset (consisting of 2488 cases) and the private Xijing_Knee dataset (consisting of 1526 cases). Notably, distinguishing between Grade 0 and Grade 3 meniscal injuries, which exhibit distinct imaging characteristics, posed relatively minimal grading challenges. Common deep learning methods typically achieve accuracies surpassing 90%, while our method attained an accuracy exceeding 92%. This underscores the robustness and effectiveness of our approach in finely grading Grade 0 and Grade 3 injuries. Nevertheless, the major challenge in automatically grading meniscal injuries comes from the identification of Grade 1 and Grade 2 injuries. The intermediary nature of Grade 2, positioned between Grades 1 and 3, results in less distinct high signal localization and intensity, contributing to grading complexities. Regardless of the methodological approach adopted, satisfactory grading outcomes for these two grades have not been achieved. Upon MRI examination, the difficulty in grading stems from the limited differentiation of high signal locations in meniscal injuries between Grades 1 and 2, leading to strikingly similar learned features that increase grading errors in deep learning algorithms when applied to test datasets. This lack of distinction poses significant challenges within the grading framework and necessitates the development of innovative techniques to achieve objective and accurate gradations.

Consequently, we propose specific methodologies to address the aforementioned challenges. Utilizing attributional attention aids in precisely pinpointing regions with distinct features, effectively focusing on these injured signals. And a quantitative method is designed to quantify the high signal intensity and the injured area of the meniscus to measure the degree of injury to the meniscus. Simultaneously, a multi-level transfer learning framework is constructed to counter the limitations posed by the meniscus’s weak signal, which restricts available information. Through multiple iterations to extract global and local features, our method can more comprehensively characterize meniscus injury features. This study not only optimized the method but also obtained multiple data sources from different institutions. The experimental data included the private Xijing_Knee dataset (1526 cases) and the public FastMRI_Knee dataset (2488 cases). Our method’s results demonstrate noticeable enhancements in grading accuracy for Grade 1 and Grade 2, respectively. These improvements significantly contribute to the overall grading accuracy, surpassing the performance of alternative methods.

To enhance the accuracy of grading diagnosis of meniscus injury, the future study will consider the multi-modal data fusion method and introduce anatomical knowledge, clinical knowledge and physiological information. In our previous analysis of factors related to meniscus injury, it was found that patient information, such as age and weight, was correlated with injury grade. Therefore, in the following studies, we believe that adding this patient information will further improve the accuracy of the grading. At the same time, the method of multi-center data joint training model will be used to improve the generalization ability of the method. Also, the development of engineering application software will be improved, and the clinical deployment will finally be realized.

**Fig. 12:**
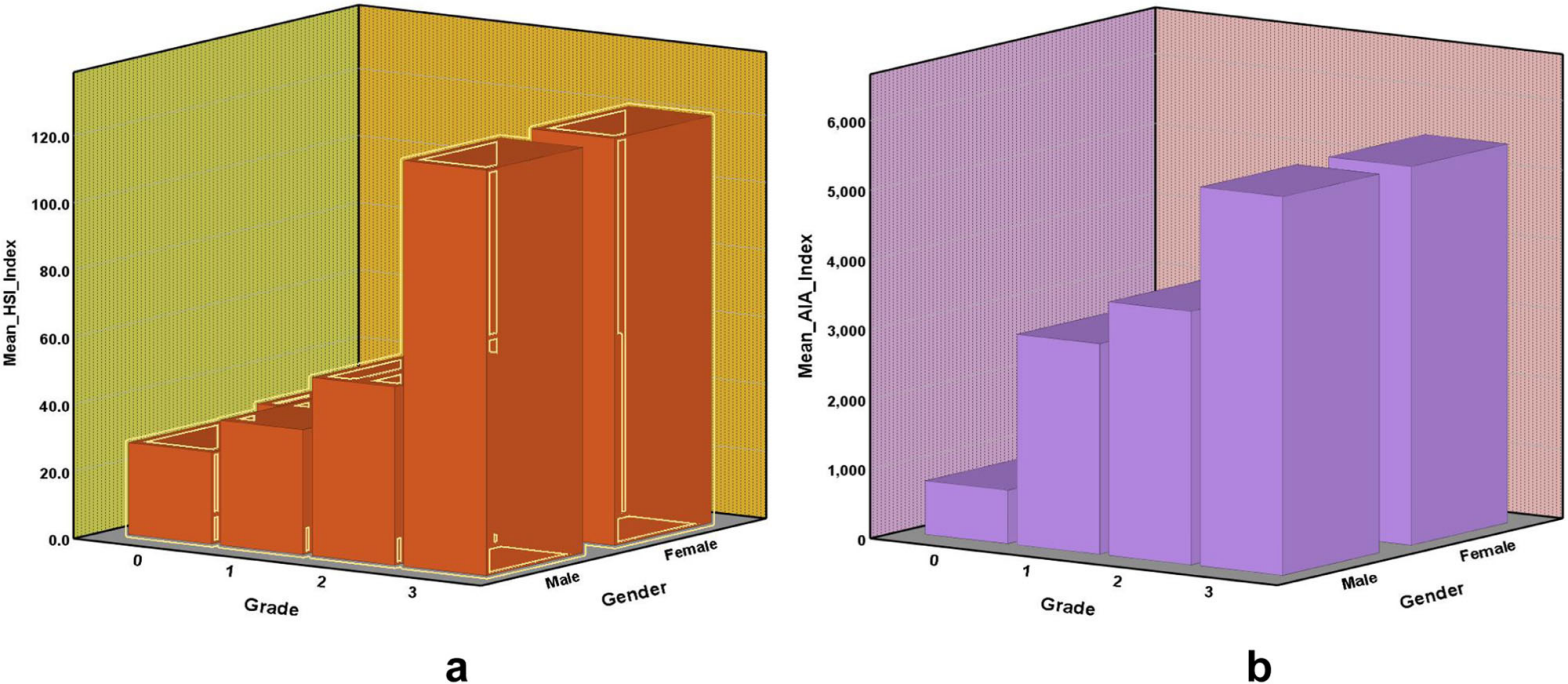
Correlation diagram of quantitative indicators of meniscus damage signal HSI and AIA with grade. In **a**, the *X*-axis is the injury grade, the *Y*-axis is the sex, and the *Z*-axis is the HSI mean value. In **b**, the *X*-axis is the injury grade, the *Y*-axis is the sex, and the *Z*-axis is the AIA mean value.

Quantitative study on the grading of meniscal injury. Human perception fails to discern subtle signal differences within images, but quantified signal values can distinctly illustrate imaging variances between healthy menisci and injured areas. In the automatic grading of meniscal injuries, we introduced a four-grade fine-grained grading method, a significant advancement from previous studies limited to binary or triple grading. Additionally, the high-low signal intensity ratio (HSI) and heat map area (AIA) of meniscus injuries were quantitatively calculated to evaluate the degree of meniscus injuries. According to the statistical analysis of the calculation results of HSI and AIA, it was found that the HSI value and AIA value in the meniscus region increased with the increase of grade, showing obvious separability, as shown in Fig. 12.

Utilizing gradation, attention heat-maps employ distinct colors to delineate the scope of potential injury, enhancing the visualization of automated diagnosis and grading of meniscal injuries, imperceptible to the human eye. The dark red area is used to represent the injury core area of the knee meniscus, which is also the core target of our grading diagnosis. The red area is the closest diffusion region to the core of injury, and the signal can be recognized by human eyes. However, the injury diffusion area represented by the three colors of yellow, green and light blue is generally difficult to distinguish, but this also reflects the difference from the normal meniscus on MRI, which is also worthy of attention in clinical diagnosis and treatment. Employing the quantitative criteria above, our method can comprehensively visualize the meniscal injury’s extent, encompassing injury location, size, and diffusion area, critically guiding clinical practices. Subsequent studies will aim to achieve a more nuanced characterization and analysis of the progressive evolution of meniscal injury, elucidating the entire system’s dynamics. This deeper insight into the mild-to-severe spectrum of meniscal injuries will facilitate the development of more effective diagnosis and treatment plans.

Clinical validation of visualization and qualitative grading. The VIFG method, reliant on automatic grading, employs visual attention heat-maps to denote injury locations. This paper presents an in-depth analysis based on clinical research. The anatomical study of the meniscus found that the distribution of blood vessels in the meniscus was evenly divided into three equal parts from the outside and inside, as shown in Fig. 11a. The outer third, termed the ‘red zone,’ enjoys robust vascularity, often facilitating self-healing without necessitating surgery for less severe injuries. The middle third, termed the ‘red-white zone,’ possesses partial vascularity with limited self-healing capabilities. Conversely, the inner third, termed the ‘white area,’ exhibits minimal to no vascularity, necessitating surgical intervention in case of injury. Recent research indicates that improper meniscectomy can result in an uneven distribution of synovial fluid within the joint, compromising its buffering capacity. This often leads to articular cartilage surface degeneration, joint instability, and significantly elevates the risk of long-term osteoarthritis. To ensure precise clinical interventions and maximal preservation of meniscal function, surgical interventions involving minimal excision or suturing should be considered for meniscal injuries. The determination of excision or suturing relies not only on the type and size of the injury but also on the vascularity at the injured site.

Currently, the selection of surgical excision areas relies heavily on surgeon expertise and intraoperative judgment. However, consensus lacks regarding the preoperative identification of blood supply influence on injuries and the precise selection of excision areas. Solely relying on knee MRI does not provide adequate information for effective decision-making. In response, this research visualized MRI imaging injury signals more intuitively through heat-maps with distinctive colors, showcasing both visible and imperceptible signals. Simultaneously, three areas corresponding to the injury site and meniscus blood supply were categorized as the red-red zone, red-white zone, and white zone. Through qualitative classification of meniscus injury and clinical case analysis, a refined classification standard was proposed, delineating Grade 2 into finer subtypes. The subdivision injury signal subtype includes: “2a: horizontal or oblique stripes with high signal located in the red-red zone”; “2b: horizontal or oblique stripes with high signal located in the red-white region”; “2c: horizontal or oblique stripes with high signal located in the white area”. The anatomic results corresponding to the high signal of meniscus in Grade 2 injury proposed in this paper have been verified in arthroscopy of dozens of clinical operations, as shown in Fig. 11b. Grade 2a, exhibiting good blood supply, might self-heal, thus considering conservative treatment. For Grade 2b, if the injury isn’t severe, it might self-recover, negating the need for surgical intervention. Conversely, Grade 2c lacks blood supply, requiring surgical removal as self-healing is unlikely. The visualization outcomes of this method complement the qualitative grading of meniscal injuries more accurately, providing guidance for clinical meniscus injury surgical planning, enhancing diagnostic precision, and improving treatment efficacy. Furthermore, it’s hoped that further research will verify and enhance the classification rules of these subtypes, yielding more acceptable and efficient outcomes for meniscal injury research.

Limitations and prospects. The findings of this study indicate that the deep learning-based image analysis method offers a partial solution to the challenging task of diagnosing and grading knee meniscal injuries. The method designed in this paper improves the accuracy of grading on the whole. The most important thing is to improve the accuracy of Grade 1 and Grade 2 which are the most difficult. However, limitations persist, such as the necessity for a more comprehensive set of evaluation indicators to guide precise clinical treatment beyond grading results alone. With regard to the automatic diagnosis and grading of meniscal injury, as Irmakci I et al. conducted research and analysis on meniscal injury^27^. The current overall results show that meniscal injury is the most challenging task among knee joint-related injuries, and further improvements in sensitivity, accuracy, and other aspects are still possible. Subsequent studies may focus on enhancing sensitivity and specificity by assessing biochemical components of menisci at different grades or incorporating additional imaging characteristics into the evaluation. Moreover, the expansion of the training dataset with more diverse patient characteristics could enhance model stability, facilitating future clinical deployment. It’s essential to note that this study solely establishes the feasibility of employing deep learning methods for knee meniscal injury assessment. Despite the great promise of the current preliminary study, large prospective validation studies are required to compare the interpretation of the meniscal injury detection system with the arthroscopically specified meniscal injury grade and associated histological examination.

## Methods

### Ethics statement

This study is approved by Xijing Hospital Affiliated to the Fourth Military Medical University. The study was non-interventional and retrospective, all participants in the study signed the written informed consent, and the knee MR images used in this data were anonymized. A sampled and desensitized example dataset was shared in the source code repository.

### Data source

For the task of automatic fine grading of knee meniscal injury, two datasets were verified. The public FastMRI_Knee dataset was from New York University^28^. The private data was from Xijing Hospital, the collaborating institution in this study, from February 2018 to March 2021, which was called Xijing_Knee. The format of raw data is Digital Imaging and Communications in Medicine (DICOM). Both datasets were collected from multiple centers and devices. Patients were scanned in feet first supine (FFS) position, and slice thicknesses included 3, 3.5, and 4 mm. The distribution details of both datasets are shown in Table 12. The FastMRI_knee dataset consisted of 2488 subjects and the Xijing_Knee dataset was studied in 1526 patients. The grades were annotated according to the Fisher standard by a radiologist and subsequently reviewed by two orthopedic doctors. Across the two datasets, the distribution of Grades 0 to 3 was as follows: 753 cases, 660 cases, 495 cases, and 580 cases; and 471 cases, 420 cases, 275 cases, and 360 cases, respectively. This study utilized sagittal and coronal T2-weighted MRI data from the aforementioned datasets, excluding patients with radiographic abnormalities, such as those who underwent joint replacement surgery. The MRI was subjected to bias field correction and size normalization with the final volume size set to 408 × 408 × 24 pixels. For training, validation, and testing the proposed VIFG method, the patients in the collected dataset were randomly grouped into three different sets according to the ratio of 3:1:1. In the preprocessing stage, the data is augmented by image rotation in various directions.

**Table 12:** Patient characteristics of the study population

		FastMRI_Knee (Public)	Xijing_Knee (Private)	*P*-value
Age		48.14 ± 17.13	37.44 ± 15.91	0.000**
Gender	Female	1365	503	0.028*
	Male	1123	1023	0.001*
Weight		80.72 ± 20.13	79.85 ± 20.19	0.024*
Leg	Right	1223	895	0.031*
	Left	1265	631	0.000**
Grade 0		753	471	
Grade 1		660	420	
Grade 2		495	275	
Grade 3		580	360	

Age is in years old, weight is in kilograms, and the rest is the number of samples. Variables were compared using Chi-square test. * Indicates that *P*-value is <0.05 and ** indicates that *P*-value is <0.001. Grade 0–Grade 3 indicates the sample distribution of the four grades in two datasets, respectively.

### Functional overview of meniscus grading system

**Fig. 13:**
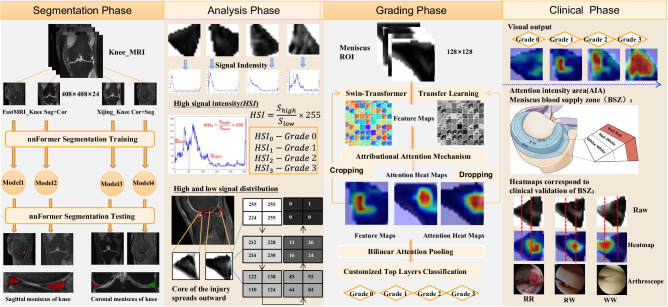
The overall framework of the functional module. The figure summarizes four aspects of this paper, the first part is the segmentation task of meniscus region from the entire knee MRI, the second part is the analysis of meniscus signals, the third part is the fine-grained grading task of meniscus injuries, and the fourth part combines its results with clinical knowledge to propose a new subtype classification and verify its results.

This study successfully integrated four key components, as depicted in Fig. 13, including rough segmentation of meniscus region, meniscal injury signal analysis, automatic fine grading of meniscal injury, visualization of meniscus injured region, subtypes of Grade 2 and clinical validation. Meniscus is a very small target in MR Images of the whole knee joint. Given the meniscus’s limited presence in MRI of the entire knee joint and the challenge in isolating specific damage within it amidst surrounding tissue, a segmentation method was employed to isolate the meniscus region, providing preprocessed data for in-depth analysis of meniscal damage. The analysis of meniscal injury signals revealed the distribution of high-signal areas across different grades of injury, showing a gradual diffusion pattern from the injury core to normal areas. The diffusion of injured signals to normal areas from the injured core in different directions is a gradual process. The intelligent grading function of meniscal injury is performed to accurately focus on the high-signal area of the injury, and the location of the injury is displayed through the heat-maps. The intelligent grading function precisely highlighted the high-signal injury area, displayed injury location via heat maps, and aligned well with clinical observations, particularly regarding the meniscus’s anatomical blood supply, which is crucial for determining clinical treatment plans. This study introduced a novel grading rule, particularly addressing Grade 2 injuries, subdividing them into more nuanced categories—2a favoring conservative treatment, while 2b and 2c leaning towards surgical intervention.

### Implement the overall technical process

**Fig. 14:**
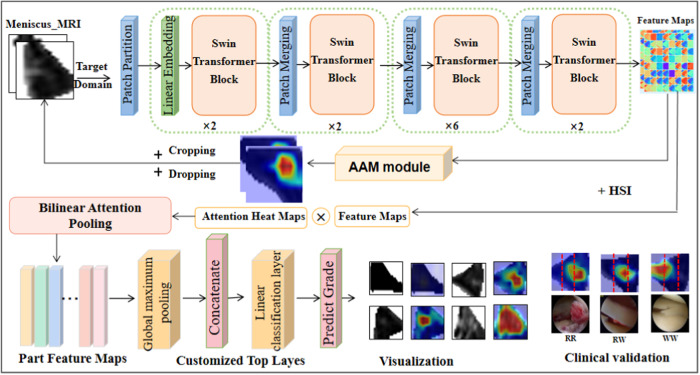
The overall technical framework diagram of the implementation details. The method diagram includes feature extraction of meniscus region using swin-transformer as backbone, and cropping operation of attention area and feedback before feature extraction. On this basis, the task of fine-grained classification is further carried out.

While realizing the aforementioned functionalities, a method rooted in attributional attention for grading meniscal injuries was developed. The comprehensive technical framework is delineated in Fig. 14. Firstly, in order to address the issue that the meniscus is a small target and the scale of observation is limited across the entire knee, MR imaging, the sagittal and coronal plane images of the knee were pre-processed, respectively. This was followed by training a coarse segmentation model specifically for meniscus area isolation. Segmentation results were cropped to enlarge the scale so as to analyze and explore the manifestations of meniscal injury in MRI. The meniscal signals from two aspects were quantitatively analyzed, including the proportion of high and low signals and the change in signal values during the extension outwards from the injured region of the core. These analyses provided detailed imaging insights into various injury grades. After the meniscus image is enhanced, the constructed multilevel transfer Swin-Transformer learning framework was used to extract distinguishable features from different levels of meniscus damage, which will be detailed in the module of the next section. The attributional attention module is then used to extract the features that make the largest contribution to the grading of identifiable regions. Once the region has been cropped and dropped, it is fed back to the feature extractor. After extracting the feature again, bilinear attention pooling is performed on the feature graph and attention graph to obtain the feature vector. Finally, the grading model is obtained through the custom grading layer at the top of the hierarchy, and the final grading result is obtained by aggregating multiple models after multiple training. Predicted labels and visual results are provided in the result output. Finally, leveraging our research and anatomical insights, Grade 2 meniscal injuries were subdivided into Grade 2 red-red, Grade 2 red-white, and Grade 2 white zones, delineating blood supply relationships to inform treatment decisions.

### Meniscus segmentation from knee MRI based on nnFormer

**Fig. 15:**
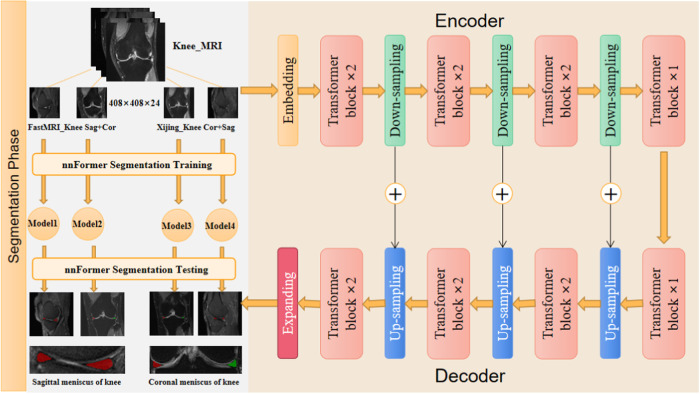
Meniscus segmentation diagram. The knee MRI data set was divided into four parts, and the two data sets corresponded to their sagittal and coronal positions, respectively. These four data sets were studied in the encoder and decoder of nnFormer, respectively, and four knee meniscus segmentation models were obtained, and meniscus regions under different perspectives were obtained in the test set.

The backbone structure of the nnFormer^29^ consists of many parts, as shown in Fig. 15. Recognizing that the convolutional network adeptly retains precise positional information and provides high-resolution low-level features, it assumes the role of Transformer blocks. Thus, the initial segment comprises a four-layer convolutional structure, primarily responsible for transforming the input image into network-manageable features. Not only does the algorithm use the combination of cross-convolution and the self-attention operation, but it also introduces a local and global volumetric self-attention mechanism for learning the volume representation. This method also proposes to replace the traditional operation of splicing or summing by skipping attention in the U-Net class structure. As this study necessitated the segmentation of the meniscus region, the nnFormer segmentation method was employed to segment this region from the entire knee MRI. Four models were individually trained on both sagittal and coronal data from the FastMRI_Knee dataset and the Xijing_Knee dataset. The resulting meniscus segmentation was tested, yielding a mean DICE index of 88.34%. Notably, the performance of this proposed method outperforms previous segmentation approaches significantly.

### Multilevel transfer swin-transformer learning framework

**Fig. 16:**
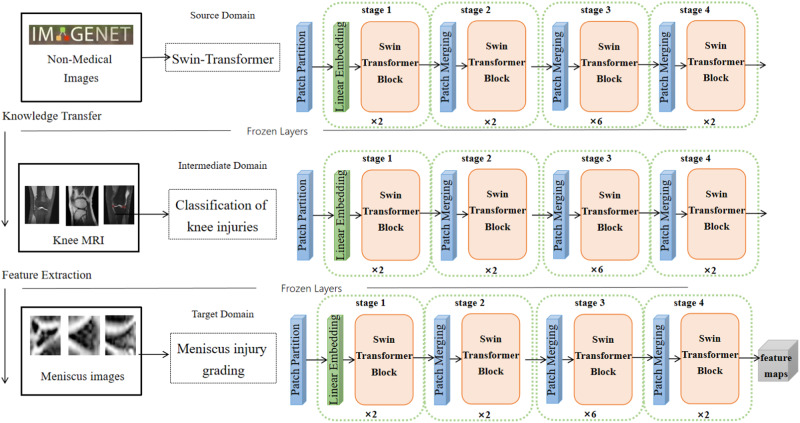
Schematic diagram of mutilevel transfer learning module. The non-medical image dataset ImageNet was used as the source domain, the middle domain was total MRI of knee joint (MRNET for short), and the target domain was MR Image data of meniscus and the backbone is swin-transformer.

Intelligent hierarchical diagnosis of meniscal injuries using deep learning encounters a challenge in constructing a network capable of fully leveraging available features. The inherent limitation of small medical image datasets necessitates innovative approaches. To maximize data utilization and acquire comprehensive information, this paper proposes a three-level transfer learning framework based on Swin-Transformer, outlined in Fig. 16. We introduce intermediate domains to reduce the domain offset difference between the source domain and the target domain on the basis of pre-training on large-scale natural images. The non-medical image data set ImageNet was used as the source domain, the middle domain was the full MRI of the knee joint, referred to as MRNET, and the target domain was MR Image data of the meniscus. Natural images can be used to learn some shallow texture information, and the high-level features of musculoskeletal and other tissues in MRI images of knee joints can be learned from the intermediate domain and then transferred to the target domain to further improve the feature extraction effect of the target domain. Adding superficial features and deep features, as well as the addition of global information on the knee joint and local information on the meniscus, is amenable to deep neural network characterization of meniscal injury. Subsequently, the attributional attention module effectively identifies fine-grained lesion features atop a foundation of diverse imaging features.

### Attributional attention module to find discriminative features

**Fig. 17:**
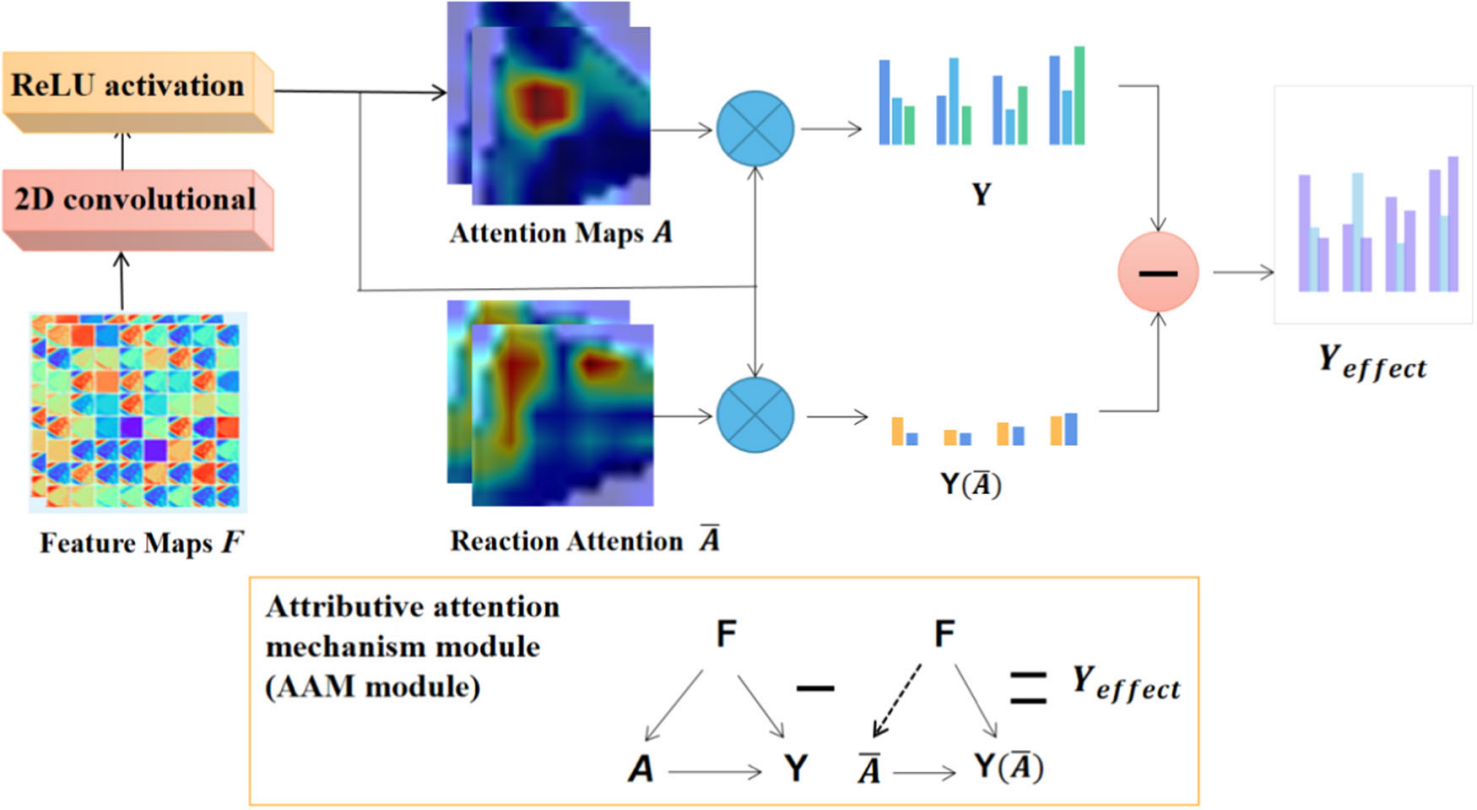
Block diagram of attributional attention module. The attribution attention module constrains the learning process, finds distinguishable features, and analyzes causal relationships between variables. To assess the quality of attention, causality was used and an attention graph with a score distribution was made.

The challenge in intelligently grading meniscal injuries resides in identifying distinct features specific to each grade. The transfer learning framework described above allows us to use the acquired swin-transform as a backbone for extracting many deep and shallow imaging features of meniscal injury. The attributional attention module constrains the learning process so as to find distinguishable features and helps us break through the black box of deep learning by analyzing the causal relationship between variables. To assess the quality of attention, the causal relation was used, producing an attention diagram with a score distribution^30^ and constructing an attributional attention network model. The purpose of this module is to discover the distinguishing features and perform a characterization of the location, shape, and diffusion severity of meniscal injury signals. The schematic block diagram of the module is shown in Fig. 17.

The features from the meniscus images were extracted through Swin-Transformer transfer learning and obtain the feature map expressed as ${\bf{F}}\in {R}^{H\times W\times N}$, where *H*, *W*, and *N* represent the feature layer’s height, width, and the number of channels, respectively. The attention module is designed to learn the spatial distribution of each part of the object, which can be expressed as an attention graph ${\bf{A}}\in {R}^{H\times W\times N}$, where *M* is the number of attention. The attention model is implemented using a two-dimensional convolutional layer and ReLU activation. The feature maps are then soft-weighted using the attention map and aggregated by the global average pooling operation $\omega$. Where * represents the multiplication of the elements of two tensors to form a global representation *h*, and these representations are strung together and normalized.

7 $${h}_{i}=\omega (F\,*\,{A}_{i})=\frac{1}{{HW}}{\mathop{\sum}\limits_{h=1}^{H}\mathop{\sum}\limits_{w=1}^{W}{\rm{F}}^{{h},{w}}{{\rm{A}}}_{i}^{h,w}},h={\rm {normalize}}([{h}_{1},{h}_{2},\ldots,{h}_{M}])$$

Inspired by the method proposed by Yongming Rao et al. ^31^, an intervention to learn to obtain visual attention by the reaction effect of grading results was used. In our practice, the intervention ${\rm {image}}(A=\bar{{\bf{A}}})$ is performed by imagining the nonexistent attention map $\bar{{\bf{A}}}$ to replace the learned attention map and keep the feature map *F* unchanged. According to Eq. (8), the final prediction *Y* after intervention $A=\bar{{\bf{A}}}$ be obtained:

8 $${\rm{Y}}({\rm {image}}({\rm{A}}=\bar{{\bf{A}}}),{\rm{F}}={\bf{F}})={\rm {class}}([\omega ({\bf{F}}\ast {\bar{{\bf{A}}}}_{M})])$$

where ${{\rm {class}}}$ is the classifier. The actual effect of the learned attention on the prediction can be represented by the difference between the observed prediction ${Y}(A={\bf{A}},X={\bf{X}})$ and its counterproductive effect

9 $${Y}({\rm {image}}(A=\bar{{\bf{A}}}),X={\bf{X}}):{Y}_{{\rm {effect}}}={E}_{\bar{{\bf{A}}} \sim \gamma }[Y({{A}}={\mathbf{{A}}}),\left.F={\bf{F}}\right)-Y({\rm {image}}(A=\bar{{\bf{A}}}),F={\bf{F}})]$$

The effect on prediction was represented as ${Y}_{{\rm {effect}},\gamma }$ is the distribution of reactive attention. Attention function is used to measure whether learning attention is focused on distinguishable area utilization tasks. The influence measurement of this characteristic is used as a supervisory signal that explicitly guides the attentional learning process. The total loss function can be expressed as:

10 $${L}_{{\rm {all}}}={L}_{{\rm {ce}}}({Y}_{{\rm {effect}}},y)+{L}_{{\rm {class}}}$$

where *y* is the grading label, *L*_ce_ is the cross-entropy loss, and *L*_class_ represents the original objective, such as standard classification loss.

### Attention guide discriminant feature augmentation

Having discovered the characteristics of meniscus separability grading using causality, augmenting the data in this area in order to fully utilize the key positional information provided by the data was developed. The grading of the four grades of meniscal injury is a fine-grained classification problem. Since the differences between grades are small, achieving accurate grading is a huge challenge. In this paper, the key step is to extract the most discriminative local features from the entire meniscus region. The intensity and location of the high signal of meniscal injury contribute specifically and decisively contribution to the determination of the grade. Thus it guides the data augmentation via the attention map acquired by the attributional attention module. The presence of attention in the network can encourage the model to pay more attention to the distinguishable area of meniscal injury by cropping and dropping. This allows for more accurate characterization of imaging features such as texture, signal intensity, and injury distribution can be more accurately characterized in the high-signal area of meniscal injury so as to obtain better grading results. Attention cropping in the network can distinguish the differences between local areas by cropping and adjusting the size of local areas so as to extract more distinctive local features. The details of the implementation are described below. Firstly, the features of the image **I** were extracted, and the feature maps were expressed as ${\bf{F}}\in {R}^{H\times W\times N}$, where $H,W,N$ represented the feature layer’s height, width and the number of channels, respectively. The attention maps is obtained by formula 11, which is expressed as ${\bf{A}}\in {R}^{H\times W\times M}$

11 $${\bf{A}}=f({\bf{F}})={{\cup }_{k=1}^{M}{\bf{A}}}_{k}$$

where $f(\cdot )$ is a function. ${{\bf{A}}}_{k}\in {R}^{H\times W}$ represents part of the signal in the meniscus region. *M* is the number of attention maps.

Data augmentation is a common processing method, but Random data augmentation is low efficient.With attention maps, data can be more efficiently augmented. For each training image, one of its attention map ${{\bf{A}}}_{k}$ was randomly choosed to guide the data augmentation process and normalize it as${k}_{{\rm {th}}}$ Augmentation Map ${{\bf{A}}}_{k}^{\ast }\in {R}^{H\times W}$.

12 $${{\bf{A}}}_{k}^{\ast }=\frac{{{\bf{A}}}_{k}-{\rm {min}}({{\bf{A}}}_{k})}{{\rm {max}}({{\bf{A}}}_{k})-{\rm {min}}({{\bf{A}}}_{k})}$$

With augmentation maps, more detailed features are extracted by resizing the image of the area. Attention cropping was used. Firstly, Crop Mask *C*_*k*_ was obtained from ${{\bf{A}}}_{k}^{\ast }$ by setting element ${{\bf{A}}}_{k}^{\ast }(i,j)$ which is greater than threshold ${\theta }_{{\rm {c}}}\in [0,1]$ to 1, and others to 0, as represented in formula 13. This region is enlarged from the original image as input data for augmentation.

13 $${C}_{k}(i,j)=\left\{\begin{array}{ll}1,{\rm {if}}\,{{\bf{A}}}_{k}^{\ast }(i,j)\, > \,{\theta }_{{\rm {c}}}\\ 0,{\rm {otherwise}}\end{array}\right.$$

To encourage the attention map to represent parts of multiple objects of recognition, attention dropping was used. Drop Mask *D*_*k*_ was obtained by setting element ${{\bf{A}}}_{k}^{\ast }(i,j)$ which is greater than threshold ${\theta }_{{\rm {d}}}\in [0,1]$ to 0, and others to 1, as represented in Eq. 7.

14 $${D}_{k}(i,j)=\left\{\begin{array}{l}1,{\rm {if}}\,{{\bf{A}}}_{k}^{\ast }(i,j)\, > \,{\theta }_{c}\\ 0,{\rm {otherwise}}\end{array}\right.$$

Since the drop operation can remove some parts from the image, the network will be encouraged to propose other differentiated parts, which means that the information can be better seen while the robustness and accuracy of the grading will be improved.

Inspired by Bilinear Pooling aggregates feature representation from two-stream network layers, Bilinear Attention Pooling (BAP) proposed to extract features from these parts^32^. It element-wise multiply feature maps **F** by each attention map **A**_*k*_ in order to generate *M* part feature maps **F**_*k*_, as shown in Eq. (15), where ⊙ denotes element-wise multiplication for two tensors.

15 $$\,{{\bf{F}}}_{k}={{\bf{A}}}_{k}\odot {\bf{F}}(k=1,2,...,M)$$

Let $\varGamma ({\bf{A}},{\bf{F}})$ indicates bilinear attention pooling between attention maps **A** and feature maps **F**. It can be represented in Eq. 16, where $g(\cdot )$ is a feature extraction function.

16 $$\varGamma ({\bf{A}}{{,}}{\bf{F}})=\left(\begin{array}{c}g({{\bf{A}}}_{1}{{\odot }}{\bf{F}})\\ g({{\bf{A}}}_{2}{{\odot }}{\bf{F}})\\ \cdots\\ g({{\bf{A}}}_{M}{{\odot }}{\bf{F}})\end{array}\right)=\left(\begin{array}{c}{f}_{1}\\ {f}_{2}\\ \cdots \\ {f}_{M}\end{array}\right)$$

After bilinear attention pooling, the feature matrix is obtained. After customized grading layers, including the maximum pooling layer of the whole play, concatenate, and linear grading layer, grading results are finally obtained. By changing the training parameters, multiple training models are obtained for aggregation prediction, and the best grading results are obtained.

### Reporting summary

Further information on research design is available in the Nature Research Reporting Summary linked to this article.

### Supplementary information

Reporting Summary

## Data Availability

The data used in this study is not open access due to privacy and security concerns. After obtaining the sharing agreement, it can be shared with third parties for reasonable use, relevant requests should be addressed to A.L. (LuoAnlin@stu.xidan.edu.cn). To enable a complete run of the code shared in this study, a minimum amount of desensitized sample data is shared with the code. The public datasets used in this study can be downloaded at https://fastmri.med.nyu.edu/.

## Code availability

Source code of this study is provided at https://github.com/LorraineAnlinLuo/VIFG_Meniscus-injury-grading.
