# Supplementary material for: Visual interpretable MRI fine grading of meniscus injury for intelligent assisted diagnosis and treatment
Source: NPJ Digit Med. 2024 Apr 15;7:97. doi: 10.1038/s41746-024-01082-z (PMC11018801; doi:10.1038/s41746-024-01082-z)
Supplement: Supplementary file 1 — Reporting Summary [file 41746_2024_1082_MOESM1_ESM.pdf]

nature portfolio

Corresponding author(s): NPJDIGITALMED-07399R1

Last updated by author(s): Feb 19, 2024

Reporting Summary

Nature Portfolio wishes to improve the reproducibility of the work that we publish. This form provides structure for consistency and transparency in reporting. For further information on Nature Portfolio policies, see our Editorial Policies and the Editorial Policy Checklist.

Statistics

For all statistical analyses, confirm that the following items are present in the figure legend, table legend, main text, or Methods section.

n/a	Confirmed	
☐	☒	The exact sample size (*n*) for each experimental group/condition, given as a discrete number and unit of measurement
☐	☒	A statement on whether measurements were taken from distinct samples or whether the same sample was measured repeatedly
☐	☒	The statistical test(s) used AND whether they are one- or two-sided<br>*Only common tests should be described solely by name; describe more complex techniques in the Methods section.*
☐	☒	A description of all covariates tested
☐	☒	A description of any assumptions or corrections, such as tests of normality and adjustment for multiple comparisons
☐	☒	A full description of the statistical parameters including central tendency (e.g. means) or other basic estimates (e.g. regression coefficient) AND variation (e.g. standard deviation) or associated estimates of uncertainty (e.g. confidence intervals)
☐	☒	For null hypothesis testing, the test statistic (e.g. *F*, *t*, *r*) with confidence intervals, effect sizes, degrees of freedom and *P* value noted<br>*Give P values as exact values whenever suitable.*
☐	☒	For Bayesian analysis, information on the choice of priors and Markov chain Monte Carlo settings
☐	☒	For hierarchical and complex designs, identification of the appropriate level for tests and full reporting of outcomes
☐	☒	Estimates of effect sizes (e.g. Cohen's *d*, Pearson's *r*), indicating how they were calculated

Our web collection on statistics for biologists contains articles on many of the points above.

Software and code

Policy information about availability of computer code

Data collection	The Python libraries of Pydicom, SimpleITK, and nibable were used for reading data and the meta information. The feature extractor is based on open source implementation of Swin-Transformer provided in https://github.com/microsoft/Swin-Transformer. The feature extraction method of radiomics comes from the resource package Pyradiomics.
Data analysis	Source code of this study is provided at https://github.com/LorraineAnlinLuo/VIFG_Meniscus-injury-grading.

For manuscripts utilizing custom algorithms or software that are central to the research but not yet described in published literature, software must be made available to editors and reviewers. We strongly encourage code deposition in a community repository (e.g. GitHub). See the Nature Portfolio guidelines for submitting code & software for further information.

Data

Policy information about availability of data

All manuscripts must include a data availability statement. This statement should provide the following information, where applicable:

- Accession codes, unique identifiers, or web links for publicly available datasets
- A description of any restrictions on data availability
- For clinical datasets or third party data, please ensure that the statement adheres to our policy

The data used in this study is not open access due to privacy and security concerns. After obtaining the sharing agreement, it can be shared with third parties for

reasonable use, relevant requests should be addressed to A.L. (LuoAnlin@stu.xidan.edu.cn). To enable a complete run of the code shared in this study, a minimum amount of desensitized sample data is shared with the code. The public datasets used in this study can be downloaded at https://fastmri.med.nyu.edu/.

Research involving human participants, their data, or biological material

Policy information about studies with human participants or human data. See also policy information about sex, gender (identity/presentation), and sexual orientation and race, ethnicity and racism.

Reporting on sex and gender	Of the 2,488 samples of FastMRI_Knee used in the study, 1,123 were from men and 1,365 were from women. Of the 1526 samples in the Xijing_Knee(Private) dataset, 1123 were from men and 1365 were from women. Gender information was not included in the study, but had little effect on meniscus injury grading.
Reporting on race, ethnicity, or other socially relevant groupings	In this study, the public data FastMRI_Knee came from European and American races, and the private data set Xijing_Knee came from Asian races.
Population characteristics	The specific basic characteristics of the population of this study object are detailed in Table 2 "Patient characteristics of the study population", including age distribution, gender distribution, left leg or right leg of the population in the two data sets.
Recruitment	The private data used in this study were MR Images of 10-80 year old patients with meniscus injury in Xijing Orthopaedic Hospital. No other specific selection criteria were included.
Ethics oversight	This study was approved and supervised by Xijing Orthopedic Hospital.

Note that full information on the approval of the study protocol must also be provided in the manuscript.

Field-specific reporting

Please select the one below that is the best fit for your research. If you are not sure, read the appropriate sections before making your selection.

☒ Life sciences ☐ Behavioural & social sciences ☐ Ecological, evolutionary & environmental sciences

For a reference copy of the document with all sections, see nature.com/documents/nr-reporting-summary-flat.pdf

Life sciences study design

All studies must disclose on these points even when the disclosure is negative.

Sample size	FastMRI_Knee(Public) 2488 cases; Xijing_Knee(Private) 1526 cases
Data exclusions	Subjects with arthroprostheses were excluded, as well as subjects with incorrect scanning posture, incomplete scanning, and poor quality knee MR Images.
Replication	The accuracy can be used to measure the performance of age estimation, and the weight parameters are also shared. Any investigator interested in this study can reproduce this study using similar data.
Randomization	When training the model, randomly sampled data sets are used, and the selection of training, verification and testing of data sets is also random. At the same time, this study also conducted a five-fold cross-validation experiment to ensure randomness.
Blinding	Data collection was blinding to participants in this study.

Reporting for specific materials, systems and methods

We require information from authors about some types of materials, experimental systems and methods used in many studies. Here, indicate whether each material, system or method listed is relevant to your study. If you are not sure if a list item applies to your research, read the appropriate section before selecting a response.

Materials & experimental systems

n/a	Involved in the study
☒	☐ Antibodies
☒	☐ Eukaryotic cell lines
☒	☐ Palaeontology and archaeology
☒	☐ Animals and other organisms
☒	☐ Clinical data
☒	☐ Dual use research of concern
☒	☐ Plants

Methods

n/a	Involved in the study
☒	☐ ChIP-seq
☒	☐ Flow cytometry
☒	☐ MRI-based neuroimaging

Plants

Seed stocks	No content
Novel plant genotypes	No content
Authentication	No content
